# Effect of Stereochemically Active Electron Lone Pairs on Magnetic Ordering in Trivanadates

**DOI:** 10.1021/acs.inorgchem.3c01760

**Published:** 2023-08-02

**Authors:** George Agbeworvi, Wasif Zaheer, John D. Ponis, Joseph V. Handy, Jaime R. Ayala, Justin L. Andrews, Parker Schofield, Cherno Jaye, Conan Weiland, Daniel A. Fischer, Sarbajit Banerjee

**Affiliations:** † Department of Chemistry and Department of Material Science and Engineering, Texas A&M University, College Station, Texas 77845-3012, United States; ‡ Material Measurement Laboratory, National Institute of Standards and Technology, Gaithersburg, Maryland 20899, United States

## Abstract

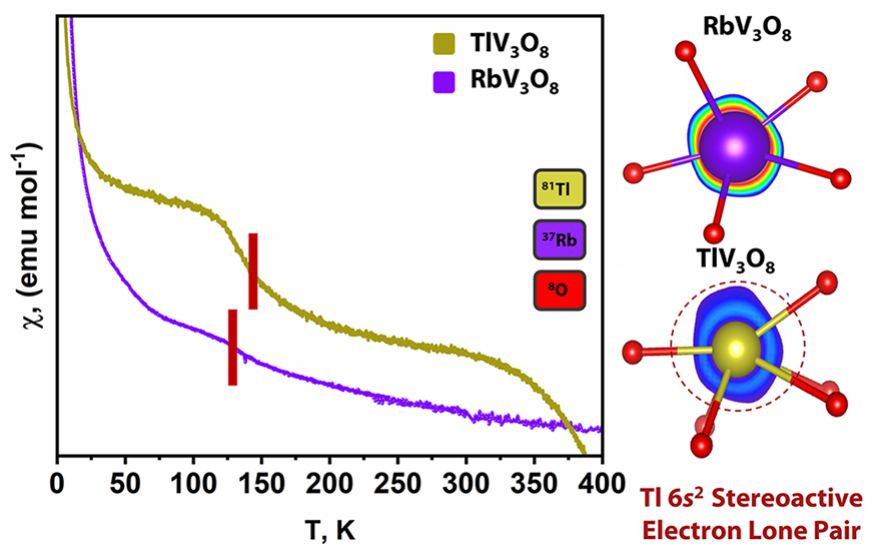

Stereoactive electron lone pairs derived from filled 5/6s^2^ states of p-block cations are an intriguing electronic and geometric structure motif that have been exploited for diverse applications such as thermoelectrics, thermochromics, photocatalysis, and nonlinear optics. Layered trivanadates are dynamic intercalation hosts, where the insertion of cations can be used to tune electron correlation, charge localization, and magnetic ordering. However, the interaction of 5/6s^2^ stereoactive electron lone pairs with layered trivanadates remains unexplored. In this study, we contrast s- and p-block trivanadates and map off-centering in the coordination environment and reduction in symmetry arising from the stereochemical activity of lone pair cations to the emergence of filled antibonding lone-pair 6s^2^–O 2p hybridized states. The former is studied by high-resolution single-crystal X-ray diffraction studies of TlV_3_O_8_ and isostructural RbV_3_O_8_ to probe distinct differences in Tl and Rb coordination environments and the resulting modulation of V–V interactions in V_3_O_8_ slabs. The latter has been probed by variable-energy hard X-ray photoelectron spectroscopy (HAXPES) measurements, which manifest orbital-specific contributions from bonding and antibonding interactions of stereoactive Tl 6s^2^ electron lone pairs in TlV_3_O_8_. The spectroscopic assignment of valence band states to stereoactive lone pairs is further corroborated by first-principles electronic structure calculations, crystal orbital Hamilton population analyses, and electron localization function maps. The presence of the Tl 6s^2^ electron lone pair in TlV_3_O_8_ brings about the off-centering of Tl^+^ cations, which leads to anisotropy in Tl–O bonds. The off-centering of Tl ions weakens V–O bonds in one direction, which subsequently strengthens directional V–V coupling. Magnetic measurements reveal ferromagnetic signatures for both RbV_3_O_8_ and TlV_3_O_8_. However, the differences in V···V interactions significantly affect the energy balance of the superexchange interactions, resulting in an ordering temperature of 140 K for TlV_3_O_8_ as compared to 125 K for RbV_3_O_8_. The results demonstrate the distinctive effects of stereochemically active lone pairs in modifying electronic structure near the Fermi level and for mediating superexchange interactions.

## Abstract 2

Stereochemically active electron lone pairs derived from p-block cations represent a versatile geometric and electronic structure motif that can be used to tune Fermi surfaces of periodic solids. In this study, we contrast s- and p-block monovalent trivanadates and demonstrate that lone-pair repulsions and concomitant lattice anharmonicity drive anisotropic structural distortions, which in turn modulates magnetic ordering of electrons on the trivanadate host framework.

## Introduction

Stereoactive electron lone pairs derived from filled 5/6s^2^ states of p-block cations represent an attractive electronic structure motif that can be finely modulated based on cation identity, interatomic separation, and stoichiometry to engender precise tuning of the energy and curvature of Fermi surfaces in solid-state compounds.^1,2^ Lattice anharmonicity induced by lone-pair repulsions on p-block cations underpins an off-centering within their coordination environment and structural transformations that have substantial implications for thermoelectrics, thermochromics, photocatalysis, and nonlinear optics.^2,3^ Positioning p-block cations with filled 5/6s^2^ electron pairs that have the potential for stereochemical activity within the interstitial sites of insertion hosts holds promise for tuning polaron localization, magnetic ordering,^3,4^ and lattice phonon structure. Vanadium oxides (V_*y*_O_*z*_) are such a versatile class of insertion hosts with considerable variation of structure types along an exceedingly “rugged” free energy landscape;^5^ these insertion hosts can accommodate s-, p-, and d-block cations across a diverse range of interstitial sites to yield M_*x*_V_*y*_O_*z*_ bronzes.^6−8^ The numerous local minima and flexibility of structure types derives from the facile accessibility of different vanadium oxidation states and the ability to stabilize tetrahedral, square pyramidal, and octahedral local coordination environments.^8,9^ Ternary vanadium oxide bronzes where the interstitial species are p-block cations are relatively underexplored beyond a few examples in the 1D tunnel-structured β-M_*x*_V_2_O_5_ (M = Pb, Sn, Tl) and 2D layered δ-M_*x*_V_2_O_5_ (M = Pb, Sn, Tl) series.^6,3,10^ Much less explored are trivanadates with the formula M_*x*_V_3_O_8_. In these compounds, monovalent or divalent ions are accommodated (with partial reduction of vanadium sites as required) between the V_3_O_8_ framework.^11,12^ As a result, electron correlation, charge localization, and magnetic ordering can be tuned in these structures as a function of structure, cation stoichiometry, and cation separation.^19,20^ In this article, we examine the effect of stereoactive lone pairs on magnetic ordering in TlV_3_O_8_ single crystals by contrasting their crystal structure, electronic structure, and magnetic properties to those of an s-block monovalent analogue, RbV_3_O_8_. As such, we delineate the strong coupling of spin, charge, lattice, and orbital degrees of freedom that underpin magnetic transitions.

As an example of the emergent behavior resulting from the strong coupling of spin, charge, orbital, lattice, and atomic degrees of freedom, BaV_3_O_8_ is best understood as a geometrically frustrated system exhibiting short- and long-range antiferromagnetic (AFM) ordering between (nominally) tetravalent vanadium ions at low temperatures.^13,14^ In contrast, NiV_3_O_8_ exhibits ferromagnetic (FM) behavior characterized by two critical ordering temperatures (2 and 27 K). However, the Curie–Weiss constant (−25 K) reveals a weak competing AFM interaction.^15^ The magnetic properties of Ag_1+*x*_V_3_O_8_ and Na_1+*x*_V_3_O_8_ show pronounced variations as a function of the cation stoichiometry, *x*. For both systems, when *x* > 0.25, the susceptibilities show a round maximum at low temperatures, whereas for *x* ≤ 0.25, they exhibit a Curie–Weiss-type dependence.^16^ Despite extensive studies of the magnetic properties of the M_*x*_V_3_O_8_ trivanadates, the effect of lone-pair interactions manifested when M is a p-block cation with filled 5/6s^2^ electrons remains to be studied. Cations with lone pairs that are stereochemically active as a result of anion hybridization as per the revised lone pair model^17^ are known to induce structural anharmonicity leading to high magnetic transition temperatures and often engendering structural phase transitions.^18,19^ For instance, in BiMnO_5_ and related systems, local structural distortions have been shown to strongly modify the magnetic transitions.^20−22^ In this article, we investigate the role of Tl 6s^2^ lone pairs in TlV_3_O_8_ and uncover structural instabilities and resulting magnetic stabilization motifs in comparison to isostructural RbV_3_O_8_ where s-block Rb ions do not possess a stereoactive lone pair of electrons.

Specifically, we contrast the effects of metal–oxygen hybridization and covalency in both compounds using energy-variant hard X-ray photoemission spectroscopy (HAXPES) and first-principles density functional theory (DFT) calculations. We conclude that the 6s^2^ lone pair drives the off-center distortion of monoclinic TlV_3_O_8_, modifying the magnetic ordering on the vanadium sublattice. Spectral interpretation and assignment of lone-pair states at the valence band edge are aided by performing HAXPES experiments at varying energies to resolve orbital contributions based on intensity dependence of HAXPES cross sections on the angular moment quantum number.^10,19,23,24^ The assignments are further corroborated by crystal orbital Hamiltonian parameter (COHP) analyses. Using a combination of single-crystal diffraction, HAXPES, and first-principles DFT calculations, we delineate the role of 6s^2^ lone pairs of Tl^+^ in determining the structural, electronic, and magnetic properties of TlV_3_O_8_.

## Experimental Section

### Synthesis of TlV_3_O_8_ Single Crystals

Single crystals of TlV_3_O_8_ were synthesized through a hydrothermal reaction. First, 0.277 g of V_2_O_5_ (Sigma-Aldrich, 99.6%) was dissolved in ca. 1 mL of an aqueous solution of 35 vol % H_2_O_2_ (ACROS Organics), resulting in a strongly exothermic reaction. After a few minutes, before the mixture dried, about 71 mL of deionized water (prepared with a Barnstead International NANOpure Diamond ultrapure water system ρ = 18.2 MΩ cm^–1^) was added, and the mixture was stirred with the aid of a magnetic stirrer. An amount of 0.023 g of TlCOOCH_3_ (Alfa Aesar, 99.995%) was dissolved in 10 mL of deionized water and added to the vanadium oxide dispersion and stirred for 10 min. The mixture was then transferred into a 125 mL poly(tetrafluoroethylene) (PTFE) vessel, which in turn was placed within a sealed stainless-steel autoclave (Parr Instruments) and heated to 210 °C for 72 h. The obtained yellow crystals were removed from suspension by filtration, washed with copious amounts of water and 2-propanol, and allowed to dry overnight in air. Given the complexity of V–O Ellingham and Pourbaix diagrams, control of pH and redox potential is imperative to arrive at the desired oxygen stoichiometries and is achieved here with the addition of H_2_O_2_.^25,26^

### Synthesis of RbV_3_O_8_ Single Crystals

Single crystals of RbV_3_O_8_ were grown according to a modified hydrothermal method.^27^ In a typical reaction, 134.3 mg of RbNO_3_ and 165.7 mg of V_2_O_5_ (Sigma-Aldrich, 99.6%) were dispersed in 16 mL of deionized water (Barnstead International NANOpure Diamond ultrapure water system, ρ = 18.2 MΩ cm^–1^) and added to a 23 mL capacity PTFE-lined stainless steel autoclave (Parr). The autoclave was added directly to an oven maintained at 250 °C for 72 h. The autoclave was removed from the oven and allowed to cool to room temperature autogenously. Bright yellow crystals were collected by vacuum filtration and washed with copious amounts of water and 2-propanol and allowed to dry overnight in air.

#### Caution

Due caution needed to be exercised when working with Tl compounds! The thallium acetate precursor, H_2_O_2_, and final products must be handled using appropriate personal protective equipment. The powders should not be breathed-in or allowed to contact skin. The supernatant from the hydrothermal reaction of TlV_3_O_8_ will also have some residual solubilized Tl species. The hydrothermal vessel should be opened in a vented fume hood, and the residual waste should be carefully labeled and disposed.

### Structural Analysis

Single-crystal X-ray data were collected on a Bruker Quest X-ray diffractometer utilizing the APEX3 software suite, with X-ray radiation generated from a Mo–Iμs X-ray tube (Kα = 0.71073 Å). All crystals were placed in a cold N_2_ stream maintained at 110 K. Following unit cell determination, extended data collection was performed using omega and phi scans. Data reduction, integration of frames, merging, and scaling were performed with the program APEX3, and absorption correction was performed utilizing the program SADABS.^28,29^ Structures were solved using intrinsic phasing, and least-squares refinement for all structures was performed on *F*^2^. Structural refinement and the calculation of derived results were performed using the SHELXTL package of computer programs and ShelXle, with Olex2 utilized to generate electron density maps from structure-factor data.^30−32^ The crystallographic information files pertaining to the new TlV_3_O_8_ and RbV_3_O_8_ structures have been deposited in the Cambridge Structural Database and are available for access with deposition numbers 2212079 and 2257899, respectively.

### Electron Microscopy

Scanning electron microscopy (SEM) images were obtained using a JEOL JSM-7500F field-emission scanning electron microscope operated at an accelerating voltage of 5 kV. Samples were prepared for SEM by dispersing powders onto carbon tape.

### Magnetic Measurements

Magnetic measurements were performed on a Quantum Design Magnetic Property Measurement System using the Quantum Design superconducting quantum interference device (SQUID) magnetometer option. Zero-field cooling (ZFC) data were collected at 0.01, 0.1, 1, 2, and 3 T in the temperature range 2–400 K. Next, the samples were cooled again with an applied magnetic field of 0.01, 0.1, 1, 2, and 3 T, and field-cooled (FC) data were recorded in the temperature range 400–2 K. Field-dependent magnetization measurements were performed at 2, 5, 10, 20, 50, 100, 200, 300, and 400 K.

### HAXPES Measurements

HAXPES measurements were performed at the National Institute of Standards and Technology beamline SST-2 of National Synchrotron Light Source II of the Brookhaven National Laboratory. Measurements at an incident photon energy of 2 keV were performed with a pass energy of 200 eV, whereas the measurements at an incident photon energy of 5 keV were collected with a pass energy of 500 eV. The data were collected with a step size of 0.85 eV, and the analyzer axis was oriented parallel to the photoelectron polarization vector. The higher excitation energy of HAXPES circumvents deleterious charging issues that are common to ultraviolet and soft X-ray photoelectron spectroscopy.^7^ Photon energy selection was accomplished by using a double Si(111) crystal monochromator. The beam energy was aligned to the Fermi level of a silver foil before measurements.

### Near-Edge X-ray Absorption Fine Structure (NEXAFS) Measurements

V L- and K-edge measurements were performed at beamline 7-ID-1 of the National Synchrotron Light Source II of Brookhaven National Laboratory operated by the National Institute of Standards and Technology. A grid bias of −300 V was used to reduce the low-energy electrons and improve the surface sensitivity. A charge compensation gun was used to avert the charging of the samples. The data were collected with a resolution of 0.5 eV for all of the plotted spectra. The partial electron yield signals were normalized to the incident beam intensity from a freshly evaporated gold mesh. The spectra were energy calibrated to the O K-edge for a standard TiO_2_ sample.

### Computational Methods

Electronic structure calculations were performed using density functional theory as implemented in the Vienna *ab initio* simulation package (VASP).^33,34^ Initial atomic positions for TlV_3_O_8_ and RbV_3_O_8_ were obtained from the crystallography data. The projected augmented wave (PAW) formalism was used to model electron–ion interactions.^35,36^ A kinetic energy cutoff of 520 eV was used for plane-wave basis restriction. Electronic exchange and correlation effects were included using the generalized gradient approximation based on the Perdew–Burke–Ernzerhof functional (GGA-PBE).^37^ A Hubbard correction of *U* = 3.25 eV was used to account for strong electron correlation in the V 3d electrons as benchmarked in a previous study.^38^ A Γ-point-centered Monkhorst–Pack reciprocal grid of 4 × 4 × 2 points was used for the relaxation of 1 × 1 × 2 supercell structures. The structures were relaxed when each Cartesian force component was less than 0.01 eV/Å unless otherwise noted. Electron localization function plots were produced by the VASP output in VESTA.^39^ The isosurfaces were was chosen to be *n* = 0.15 for both TlV_3_O_8_ and RbV_3_O_8_.^40^ COHP analyses were performed using the software package Local Orbital Suite Toward Electronic-Structure Reconstruction (LOBSTER).^41,42^ Bunge’s description for the local basis functions was used for projection calculations with 2s and 2p orbitals for oxygen; 3p, 3d, and 4s for vanadium; 4d, 5s, and 5p for rubidium; and 5d and 6s for thallium. The absolute charge spilling is lower than 3.05% in all cases.

## Results and Discussion

**Figure 1:**
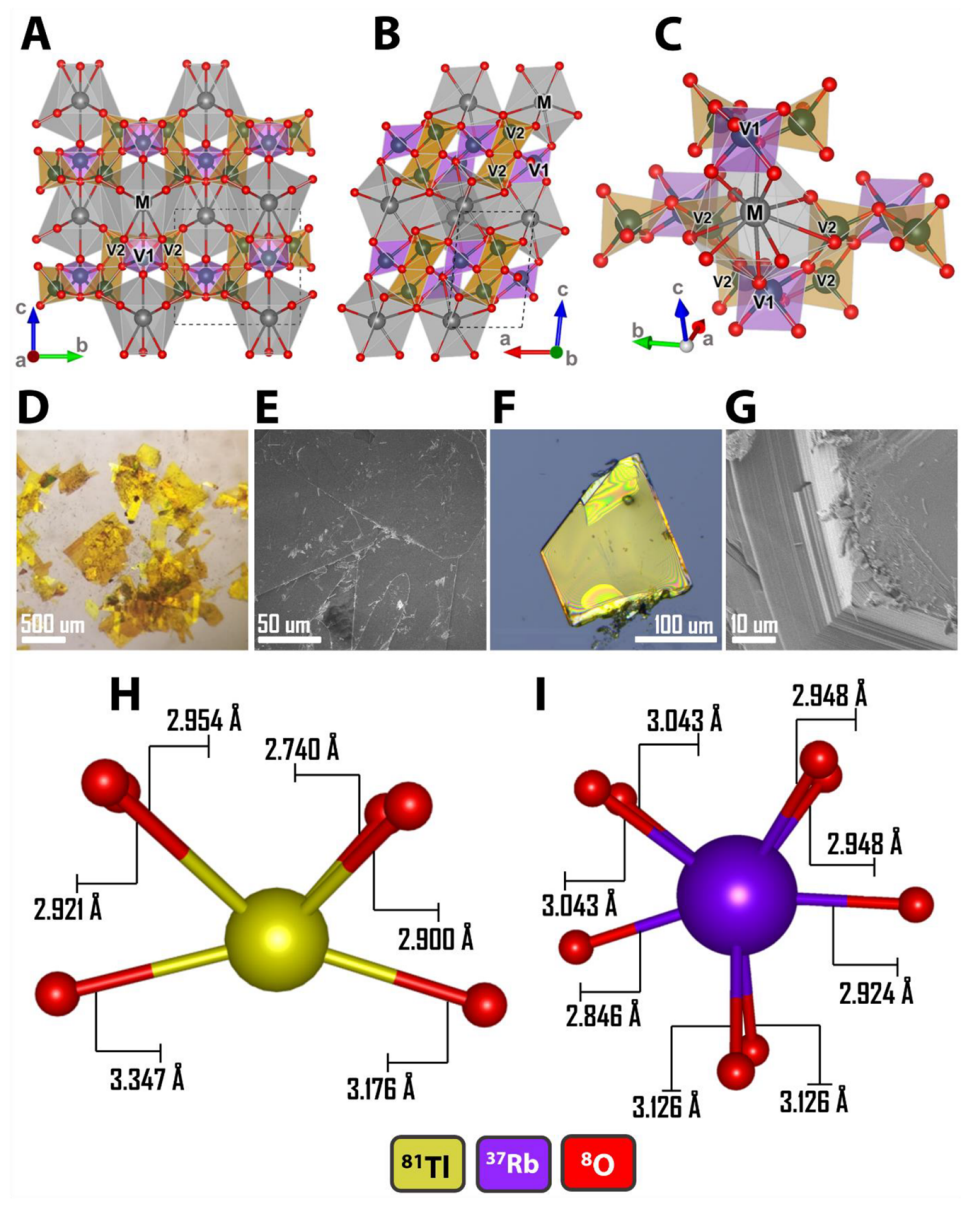
Structural characterization of MV_3_O_8_ (M = Tl, Rb) from single crystal X-ray diffraction. (A, B) Extended crystal structure of MV_3_O_8_ (M = Tl, Rb) viewed down the (A) *a*-axis and (B) *b*-axis. The intercalating cation M and inequivalent vanadium atoms (V1 denotes octahedrally coordinated; V2 denotes square-pyramidally coordinated vanadium atoms) are labeled. (C) Perspective view of the irregular 10-coordinated site in MV_3_O_8_ with respect to the surrounding V**1**O_6_ and V**2**O_5_ polyhedra. Optical and SEM images of (D, E) TlV_3_O_8_ and (F, G) RbV_3_O_8_ faceted and colored single crystals. Comparison of (H) Tl–O and (I) Rb–O coordination environments illustrating the pronounced change in local coordination environment as a result of Tl^+^ 6s^2^ stereochemical lone pairs.

MV_3_O_8_ (M = Tl, Rb) compounds with monovalent cations are isostructural and crystallize as KV_3_O_8_-type structures with *P*2_1_/*m* space group symmetry (RbV_3_O_8_, *a* = 4.9729(4) Å, *b* = 8.4096(7) Å, *c* = 7.8183(6) Å; TlV_3_O_8_, *a* = 4.9699(10) Å, *b* = 8.3814(2) Å, *c* = 7.7318(2) Å). Figure 1 shows the brightly colored single crystals and a view of the extended crystal structure obtained from solution crystals to single-crystal X-ray diffraction. Tables S1–S4 show the crystal data and structure refinement statistics for TlV_3_O_8_, and Tables S5–S8 show the crystallographic data and atomic coordinate parameters for RbV_3_O_8_. Zigzagging units of distorted VO_6_ octahedra are edge-shared with VO_5_ square pyramids to form infinite layers in the *ab*-plane. Guest cations are accommodated in large 10-coordinated interstitial sites (Figure 1). As shown in Figure 1C, this site is both face- and corner-sharing with the VO_6_ octahedra (V1), but only edge-sharing with the VO_5_ square pyramid (V2). This layered motif is reflected in the platelike habit of the single crystals (Figure 1D,F). The layered morphology of the structure is also apparent in SEM images for TlV_3_O_8_ and RbV_3_O_8_ shown in Figures 1E, S1C,D and Figures 1G, S1A,B, respectively. The Tl/Rb, V, and O elemental maps shown in Figures S2 and S3 indicate a homogeneous spatial distribution of elements in the synthesized compounds. TlV_3_O_8_ has a unit cell volume of 320.0 Å^3^, whereas RbV_3_O_8_ has a volume of 325.3 Å^3^ (contrasting Tables S1 and S5). This difference is mainly attributable to the slight stretching of the *c* lattice parameter in RbV_3_O_8_ (7.82 Å compared to 7.73 Å in TlV_3_O_8_) resulting from the V_3_O_8_ layers being pushed apart by the larger ionic radius of Rb (163–172 pm in high coordination environments as compared to 159–170 pm for Tl^+^).^43^

Figure 1H,I illustrates the local coordination environments around the monovalent cations in TlV_3_O_8_ and RbV_3_O_8_. Tl^+^ and Rb^+^ cations occupy positions between the V_3_O_8_ layers, in the proximity of 10 neighboring oxygen atoms that constitute VO_6_ and VO_5_ polyhedra (Figure 1C), thus formally establishing a bicapped square antiprismatic local coordination environment. However, the long Tl–O (3.347 Å) and Rb–O (3.343 Å) bonds (Figure 1H,I) are weakly coordinated, and as such, Tl and Rb are 6-fold and 8-fold coordinated, respectively. In TlV_3_O_8_, two of the oxygen atoms make up one edge of the VO_5_ square pyramid base, and one of the oxygen atoms forms a corner of the VO_6_ distorted octahedral base. Alternatively, in RbV_3_O_8_, two oxygen atoms each occupy the corners of the VO_5_ and VO_6_ base. As shown in Figure 1H,I, Tl···O bond distances vary from 2.740 to 3.176 Å, whereas Rb···O bond distances vary from 2.846 to 3.126 Å. The Tl^+^ cations in TlV_3_O_8_ display stereochemical activity with a significant shift of the Tl position from the center of the 10-coordinated site, as demonstrated by the change in Tl^+^–O interatomic distances and a reduction of the local symmetry.

In RbV_3_O_8_, the V(1,2)–O distances vary from 1.602 to 2.260 Å (average 1.904 Å), whereas in TlV_3_O_8_ the V(1,2)–O distances vary from 1.608 to 2.270 Å (average 1.882 Å) (comparing Tables S4 and S8). The differences in unit cell volumes of the two compounds thus arise primarily from the differences in ionic radii of the monovalent cations. Bond valence sum (BVS) calculations^44,45^ of V1 and V2 ensconced within VO_6_ polyhedra and VO_5_ square pyramids are 4.83 and 4.75, respectively, for TlV_3_O_8_, in comparison to 4.90 and 4.82 for RbV_3_O_8_. These values are consistent with previous reports of M_1+*x*_V_3_O_8_ (M = Li, Na, Ag),^16^ as well as TlV_3_O_8_,^46^ CsV_3_O_8_, KV_3_O_8_, and RbV_3_O_8_,^27^ where V1 and V2 are considered to be nearly pentavalent. HAXPES analysis of the V 2p_1/2_ and 2p_3/2_ core levels for TlV_3_O_8_ and RbV_3_O_8_ shown in Figure S4 also corroborates the idea of a modest reduction of vanadium centers. The fits show a slight reduction of vanadium centers from V^5+^ to V^4+^ formal oxidation states for both RbV_3_O_8_ and TlV_3_O_8_ as plotted in Figures S4B and S4C, respectively. The corresponding fitting details are provided in Tables S9 and S10. In RbV_3_O_8_, 12.1% of the total vanadium centers are reduced with the intercalation of Rb^+^ cations, whereas Tl^+^ intercalation reduces 11.5% of the vanadium centers in TlV_3_O_8_.

HAXPES measurements have been performed for both RbV_3_O_8_ and TlV_3_O_8_ at 2.0 and 5.0 keV to understand the differences in electronic structure manifested from the observed variations in site symmetry and atomistic structure. The valence band of V_3_O_8_ primarily comprises the O 2p and V 3d states; however, filled 5/6s^2^ states are also expected to be observed at the valence band edge in TlV_3_O_8_. The relative photoionization cross sections of orbitals decay quite rapidly as a function of incident photon energy, which is reflected in diminishing intensity of valence band spectra. However, the photoionization cross sections of subshells show a pronounced dependence on the orbital angular momentum quantum number;^17,10^ as such, the cross sections of states with substantial d*-* and f*-*orbital contributions decay much more rapidly as a function of incident photon energy as compared to s- and p-derived states.^47^ As a result, HAXPES spectra accessible at synchrotron facilities enable quantitative assessment of the orbital contributions of states at the valence band edge; stereochemically active lone pair states derived from filled 5s/6s^2^ orbitals of p-block cations can thus be selectively spotlighted by contrasting variable-energy HAXPES spectra.^6,17,10,19^

**Figure 2:**
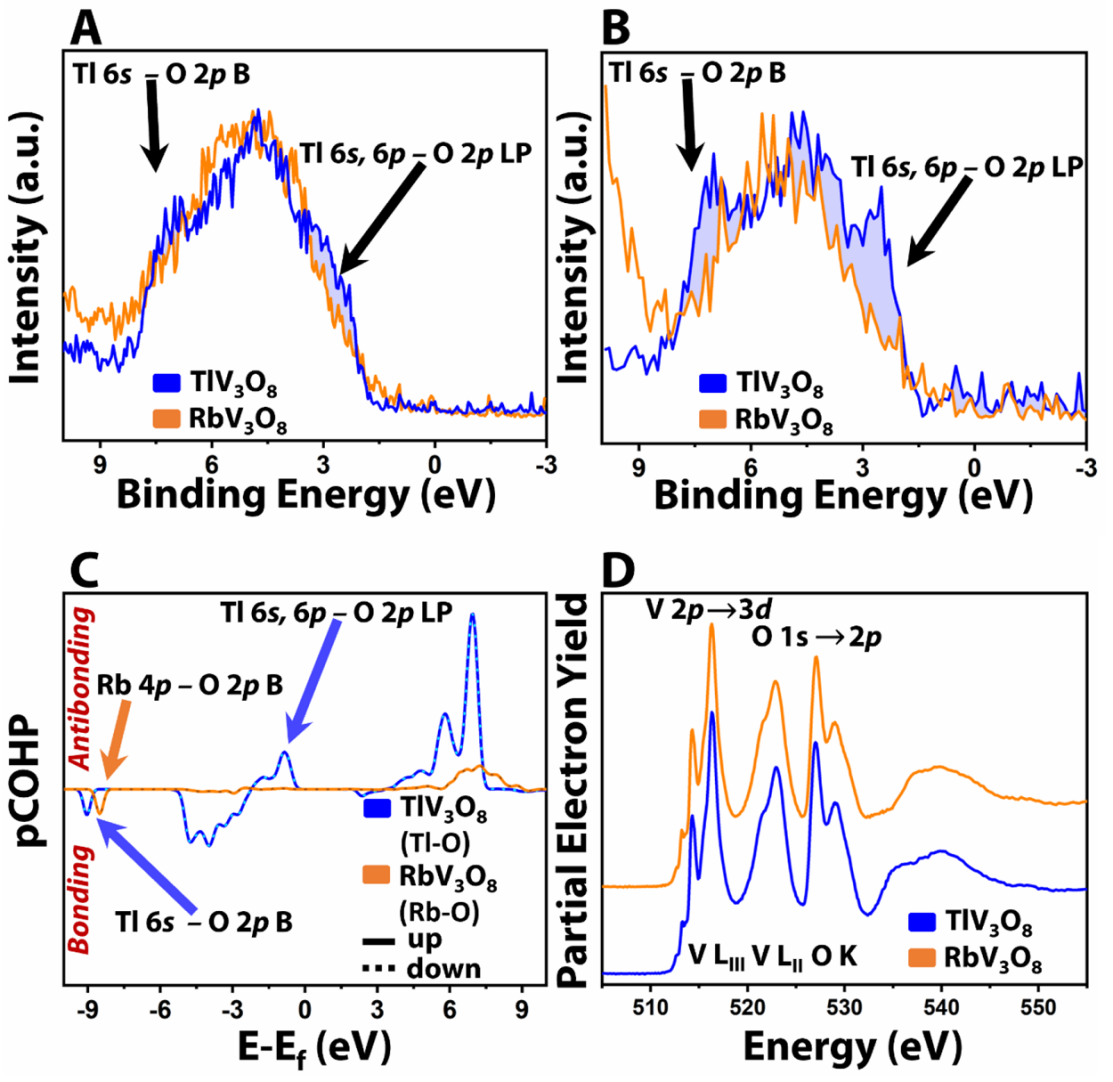
Electronic structure of RbV_3_O_8_ and TlV_3_O_8_. Overlay of HAXPES data collected for RbV_3_O_8_ and TlV_3_O_8_ at incident photon energies of (A) 2.0 and (B) 5.0 keV. (C) COHP analysis for Tl–O interactions in TlV_3_O_8_ and Rb–O interactions in RbV_3_O_8_. Interactions are plotted for comparison to HAXPES data; bonding interactions between two species are negative along the *y*-axis, whereas antibonding interactions are positive along the *y*-axis. Interactions with spin-up character are plotted using a solid line, whereas interactions with spin-down character are represented as dotted lines. (D) V L- and O K-edge NEXAFS spectra for RbV_3_O_8_ and TlV_3_O_8_.

HAXPES spectra collected at 2.0 keV for RbV_3_O_8_ and TlV_3_O_8_ are overlaid in Figure 2A. A direct comparison of valence band HAXPES spectra for RbV_3_O_8_ and TlV_3_O_8_ shows two key distinctions. First, a feature centered at a binding energy of ca. 8 eV is observed for TlV_3_O_8_ with no similar feature for its s-block counterpart, RbV_3_O_8_. Second, more electronic states can be observed at the valence band maximum of TlV_3_O_8_ as compared to RbV_3_O_8._Figure 2B plots the valence band HAXPES spectra for RbV_3_O_8_ and TlV_3_O_8_ collected at 5.0 keV. Notably, the relative intensity of the two distinctive features identified in Figure 2A increases as a function of incident photon energy, which suggests that they have pronounced 6s^2^ contributions and are derived from bonding and antibonding Tl–O interactions.^17,10,19^

To decipher the spectral difference in RbV_3_O_8_ and TlV_3_O_8_, total and atom-projected density of states (DOS) have been calculated using the GGA + *U* method by first relaxing the structures deduced from Rietveld refinements. Figure S5 compares the total DOS and atom-projected DOS for RbV_3_O_8_ and TlV_3_O_8_. The conduction band minimum for both RbV_3_O_8_ and TlV_3_O_8_ comprises electronic states derived from V 3d and O 2p states. However, the valence band maximum (VBM) of TlV_3_O_8_ shows electronic states arising from Tl orbitals, whereas such states are not observed at the VBM of RbV_3_O_8_. The presence of Tl states at the VBM indicates the role of stereochemically active lone pairs in defining the Fermi surface of TlV_3_O_8_. COHP analyses were further performed for RbV_3_O_8_ and TlV_3_O_8_ to aid with spectral interpretation and to delineate the extent and mode of hybridization between cation–anion pairs.

COHP provides insights into relative energy positioning and the pairwise bonding–antibonding character of electron lone-pair states resulting from the interaction of electron lone pairs of p-block cations with anion p states. Figure 2C plots the COHP analyses for Rb–O interactions in RbV_3_O_8_ and Tl–O interactions in TlV_3_O_8_. The COHP plots for Rb–O and Tl–O interactions in Figure 2C can be directly compared to the HAXPES data in Figure 2A,B. The presence of distinct features in the valence band of TlV_3_O_8_ and the increases in their relative intensity as a function of incident photon energy can be explained by invoking the revised lone pair model.^48,17,49^ The hybridization of Tl 6s stereoactive lone pair states with O 2p states in TlV_3_O_8_ leads to the formation of Tl 6s–O 2p bonding (B) at ca. 8 eV and Tl 6s–O 2p antibonding (AB) states at the VBM. The hybridization of the lone pair *n*s^2^ states with ligand p states is stabilized by a second-order Jahn–Teller distortion.^17^ The break in symmetry allows further hybridization of Tl 6s–O 2p AB states with unoccupied Tl 6p states in the conduction band, which leads to the formation of occupied antibonding states labeled as Tl 6s, 6p–O 2p lone pair (LP) states. Because the Tl 6s, 6p–O 2p LP states are derived from orbitals with low angular momentum, their spectroscopic signatures are more readily distinguishable at higher incident X-ray energies, as evident from Figure 2B. In Figures 2A and 2B, the Tl 6s–O 2p B and Tl 6s, 6p–O 2p LP states are represented by shaded regions centered at ca. 8 and 2 eV binding energies, respectively.

NEXAFS measurements have been acquired at the V L-edge (2p → 3d) and the O K-edge (1s → 2p) to map the conduction band of RbV_3_O_8_ and TlV_3_O_8_. The V L_III_-edge is characterized by sharp features corresponding to transitions from V 2p core states to V 3d states split by crystal field splitting in the distorted octahedral (V**1**O_6_) and square-pyramidal (V**2**O_5_) coordination geometries.^50−52^

The fine structure reflects the t_2g_ and e_g_ manifolds, which are further split as a result of partial occupancy of V 3d states and lattice distortion resulting from vanadium reduction. The V L_II_-edge cannot be interpreted solely in terms of electronic structure alone because of spectral broadening derived from the Coster–Kronig Auger decay processes. The O K-edge comprises transitions from O 1s core states to O 2p states hybridized with V 3d states that are split by crystal-field splitting. The fine structure represents the transitions to π- and σ-bonded hybrid states.^53^ Due to similar VO_5_ square pyramids and distorted VO_6_ octahedra observed in RbV_3_O_8_ and TlV_3_O_8_ in Figure 1A–C and the V 3d and O 2p contribution to conduction band states, V L-edge NEXAFS plots for RbV_3_O_8_ and TlV_3_O_8_ (Figure 2D) exhibit similar spectral features.

**Figure 3:**
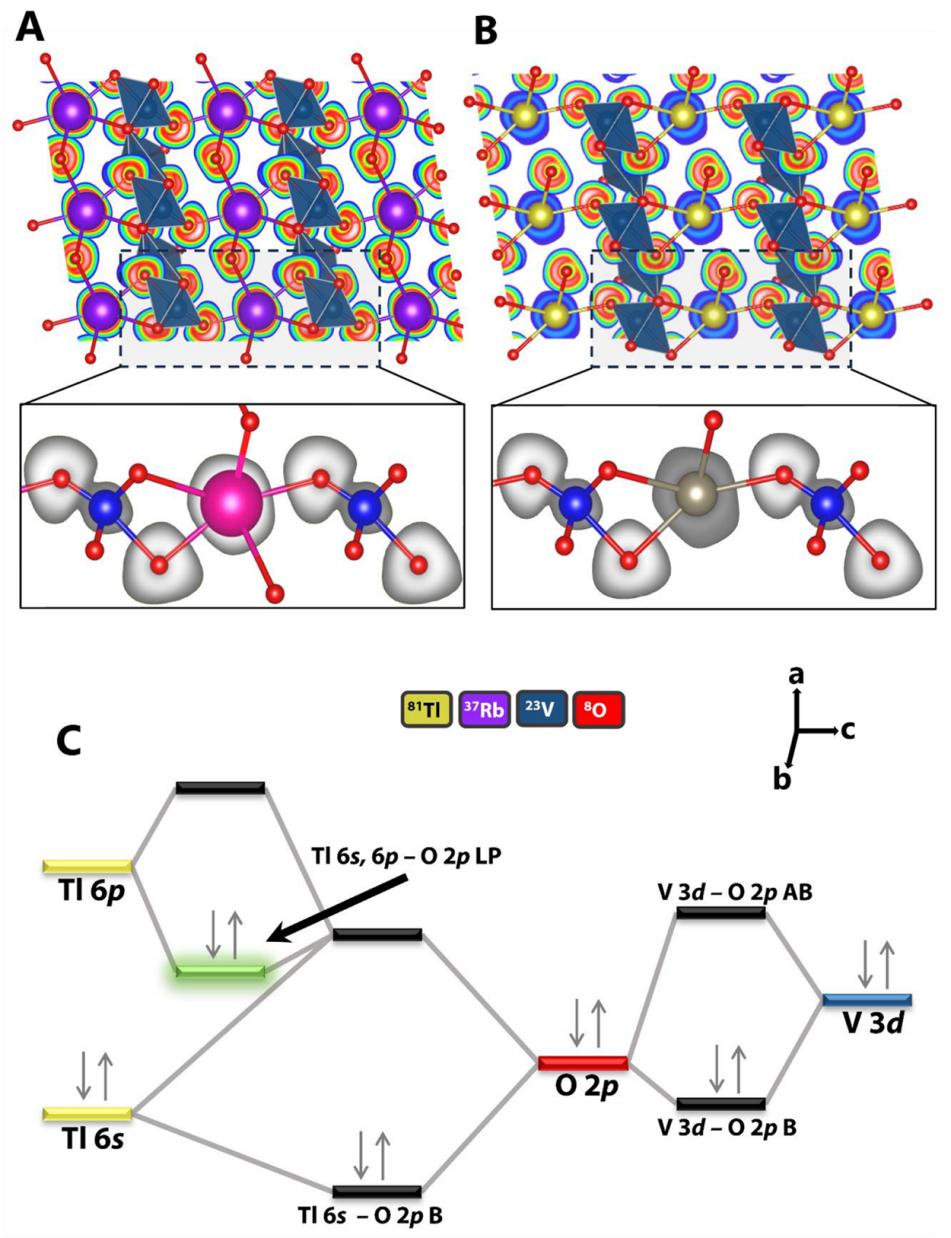
Stereochemically active lone pairs and their effects on the local electronic structure. (A) ELF slices along the (010) plane of RbV_3_O_8_. (B) ELF sliced along the (010) plane of TlV_3_O_8_. A more pronounced distortion of the ELF around the Tl center is observed, especially in the direction where the oxygen atoms are farthest from the Tl centers, reflecting the directionality of the localized stereochemically active lone pairs. (C) Molecular orbital depiction of the electronic structure of TlV_3_O_8_.

To visualize the differences in the electronic and atomistic structures of RbV_3_O_8_ and TlV_3_O_8_, electron localization function (ELF) maps have been calculated and are plotted in Figure 3. As shown in the inset of Figure 3A, the electron localization is uniform across the Rb center and reflects its positioning near the center of the interstitial site, where it is coordinated by oxygen atoms from adjacent VO_5_ square pyramids and VO_6_ octahedra. However, in the inset of Figure 3B the electron localization around the Tl center can be seen to be protruding outward precisely where the oxygen centers are the farthest from the Tl atom. The greater distortion of the ELF in TlV_3_O_8_ thus reflects strong directionality, confirming the combined steric and electrostatic effects of localized stereochemically active lone pairs. Notably, while the anisotropic distortion is clearly discernible, distinctive lone-pair lobes are not as prominent given the high coordination number of Tl centers, which manifests substantial modification of the lone-pair density as a result of repulsion with adjacent bonding pairs.^6,19^ The ELF plots further confirm that the reduction in local symmetry, off-centering, and long Tl–O interactions observed in Figure 1 can be directly attributed to the presence of stereochemically active 6s^2^ lone pairs of Tl-ions in TlV_3_O_8_, which are absent in isostructural RbV_3_O_8_. The presence of lone pairs indeed further manifests in the geometric structure where the anisotropic atomic displacement parameters (ADPs) are more pronounced for Tl compared to Rb as listed in Tables S3 and S7, with Tl having larger ADPs compared to Rb, which suggests greater disorder and distortion of the crystal lattice.

Figure 3C sketches a molecular orbital diagram to summarize the electronic structure of TlV_3_O_8_ as mapped through HAXPES and COHP. The hybridization of Tl 6s with O 2p leads to the formation of Tl 6s–O 2p and Tl 6s–O 2p AB states. The structural distortion and reduction in symmetry due to the Tl 6s electron lone pair further permit the hybridization of Tl 6s–O 2p AB states with unoccupied Tl 6p states in the conduction band. This stabilizes Tl 6s, 6p–O 2p LP states that have antibonding character and are present at the valence band maximum. The highest occupied molecular orbital (HOMO) states are highlighted in green in Figure 3C. The V 3d states hybridize with the O 2p states forming V 3d–O 2p B and AB states.

The unoccupied V 3d–O 2p AB states in the conduction band are the lowest unoccupied molecular orbital (LUMO) states in the electronic structure of TlV_3_O_8_ and are indeed probed in both V L- and O K-edge NEXAFS measurements. The key differentiating lone-pair-derived electronic structure feature of TlV_3_O_8_ is thus at the VBM and deeper within the valence band.

**Figure 4:**
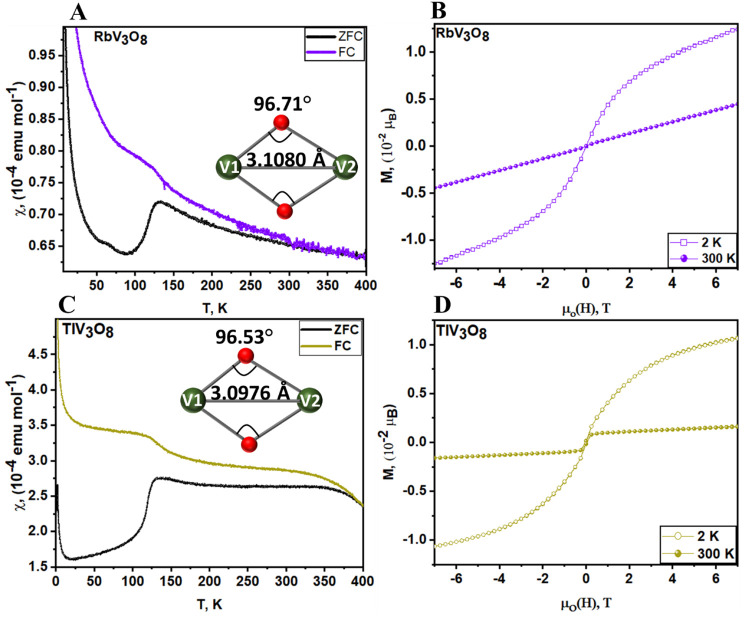
Magnetic properties of MV_3_O_8_ (M = Rb, Tl). ZFC–FC magnetic susceptibility of (A) RbV_3_O_8_ and (C) TlV_3_O_8_ at μ_0_(H) = 0.01 T. The insets in (A) and (C) depict possible magnetic superexchange interaction paths. Magnetization versus magnetic field curves plotted at 2 and 300 K for (B) RbV_3_O_8_ and (D) TlV_3_O_8_. No hysteresis is observed at 2 K.

The temperature dependence of the magnetic susceptibility, χ(*T*), of RbV_3_O_8_ and TlV_3_O_8_ has been measured in the range 2–400 K under varying external magnetic fields (Figures 4A,C and S6A,B). Ferromagnetic transitions with Curie temperatures (*T*_C_) of 125 and 140 K at 100 Oe (Figure 4A,C) are observed for RbV_3_O_8_ and TlV_3_O_8_, respectively, with the (*T*_C_) suppressed at higher applied magnetic fields (Figure S6A,B). The field-dependent behavior points to the intrinsic nature of the ferromagnetism exhibited by these compounds. At 400 K, the susceptibility of both samples increases significantly up to 350 K, followed almost immediately by an abrupt split of the zero-field cooled (ZFC)–FC (field cooled) curves upon cooling. This divergence between the ZFC–FC susceptibilities below 350 K is an indication of magnetic anisotropy in these materials. The low-temperature increase in susceptibilities observed at 50 and 25 K in RbV_3_O_8_, and TlV_3_O_8_, respectively, is most likely a result of isolated spins.^54,55^ Alternatively, the feature could possibly arise from ferromagnetic exchange coupling between canted-spin V^*n*+^ centers.^56^ The observed differences in the magnetic transition temperatures with the different cations can be attributed to differences in V···V bond distances, which ultimately reflect distortions of local structure resulting from the presence of the 6s^2^ lone-pair in Tl^+^ cations. The off-centering engendered by Tl 6s^2^ lone pair states results in the elongation of a pair of Tl–O bonds and, conversely, strengthens Tl–O interactions on the opposite side of the interstitial cation site. The directional off-centering of Tl ions weakens V–O interactions along this direction, which brings adjacent vanadium atoms into closer proximity. V1—V2 distances (separated by oxide anions) are 3.0976(7) Å for TlV_3_O_8_ and 3.1080(9) Å for RbV_3_O_8_. The shorter V1–V2 bond distance, which gives rise to a higher ordering temperature of 140 K, is a result of the subtle distortion of the Tl bonding geometry caused by the lone electron pair on the Tl^+^ cation, leading to a more asymmetric distribution of Tl–O bond lengths (Figure 1H) as well as shortening of the V1–V2 interactions (Tables S4 and S8). Based on examination of the crystal structure, the V(1)^4.83+^–O3–V(2)^4.75+^ and V(1)^4.90+^–O4–V(2)^4.82+^ superexchange interactions with bond angles of 96.53(10)° and 96.71(9)° for TlV_3_O_8_ and RbV_3_O_8_, respectively, are expected to be the most relevant. The lone pair distortion thus modulates the oxide-mediated superexchange between the square pyramidal and octahedral chains.

The ferromagnetic-like character of RbV_3_O_8_ and TlV_3_O_8_ has been further confirmed by field-dependent measurement of the magnetization measured at several temperatures under an applied magnetic field ranging from–7 to +7 T (Figure 4B,D). *M*–*H* curves for the two samples at 2 K display the typical S-shaped characteristic of ferromagnetic materials and show negligible hysteretic behavior. The isotherms of RbV_3_O_8_ above the transition temperature (Figure S7) are not linear, except at 300 K (Figure 4B), which are consistent with paramagnetic behavior at higher temperatures (300–400 K). In contrast, the isotherms of TlV_3_O_8_ at 300 K (Figure 4D) are linear only above 1 T with no tendency toward saturation, suggesting paramagnetic behavior. The slight nonlinearity in magnetization below 1 T is attributable to trace ferromagnetic impurities.

The susceptibility of RbV_3_O_8_ above 300 K has been fitted to a modified Curie–Weiss law (χ = χ_0_ + *C*/(*T* – θ)) (see Figure S8). The observed slope reflects an effective moment of μ_eff_ = 0.38 μ_B_/fu, which corresponds to 0.127 μ_B_/V atom. This value is not consistent with the theoretical effective moment expected for a d^1^ (1.73 μ_B_) system, indicating relatively minimal reduction of the vanadium centers. In addition, the intercept on the temperature axis yields a positive Weiss constant Θ of 130 K, indicating ferromagnetic nearest-neighbor dominant interactions between V atoms in the paramagnetic regime. In contrast, the susceptibility of TlV_3_O_8_ above *T*_c_ has not been fitted to a Curie–Weiss dependence as a result of the ferromagnetic impurities above *T*_c_.

**Figure 5:**
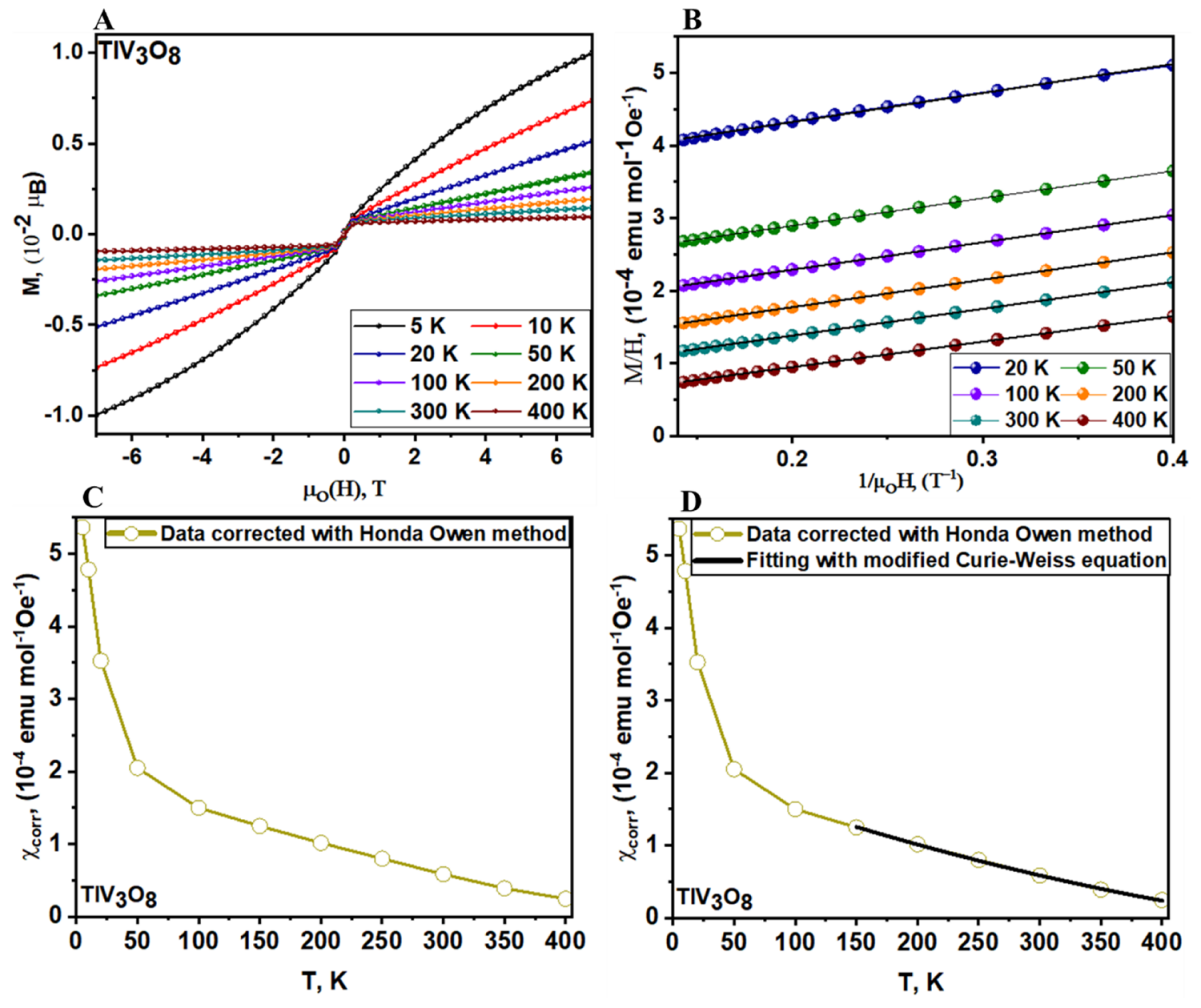
(A) Field-dependent magnetization of TlV_3_O_8_ between −7 and 7 T at representative temperatures. (B) Field-dependent magnetization data (M/H) are plotted as a function of 1/*H* at higher applied fields according to the Honda–Owen method. (C) Corrected paramagnetic susceptibility χ_corr_ extracted from isothermal magnetization curves by the Honda–Owen method. (D) Data corrected with the Honda–Owen method were fitted with the modified Curie–Weiss equation.

To determine the intrinsic magnetic susceptibility, χ, of TlV_3_O_8_, the field dependence of magnetization at varying temperatures has been measured and is shown in Figure 5A. The contribution of ferromagnetic impurities is subtracted according to the Honda–Owen method^57,58^ obtained by linearly extrapolating the M/H vs 1/*H* curves to infinite field (1/*H* → 0) as displayed in Figures 5B and S9. The concentration of the ferromagnetic impurity is estimated to be 0.00025 wt %. Figure 5C shows the temperature dependence of χ_corr_ extracted from the Honda–Owen fitting. The intrinsic magnetic susceptibility (χ_corr_) for TlV_3_O_8_ was fitted to the modified Curie–Weiss equation (χ = χ_0_ + *C*/(*T* – θ)) as indicated in Figure 5D.

The effective moment per V atom, estimated from the Curie constant, *C*, yields 0.37 μ_B_/per fu, which corresponds to 0.123 μ_B_/V atom. This effective magnetic moment like in RbV_3_O_8_ is lower than the effective moment for a d^1^ (1.73 μ_B_) system, indicating only minimal reduction of the vanadium (+5) centers. Nevertheless, it is consistent with the low values of magnetic susceptibility of magnetization (shown in Figures 4D and 5A) as well as the HAXPES plot shown in Figure S4. In addition, the intercept on the temperature axis yields a positive Weiss constant Θ of 142 K, indicating dominant ferromagnetic nearest-neighbor interactions between V atoms in the paramagnetic region.

## Conclusions

High-quality single crystals of ternary vanadates TlV_3_O_8_ and RbV_3_O_8_ with Tl^+^ and Rb^+^ ions intercalated between (V_3_O_8_)_*n*_ layers have been grown by hydrothermal synthesis. The Tl^+^ and Rb^+^ ions reside within a formally bicapped square antiprismatic interstitial site. The filled 6s^2^ lone pair on the Tl^+^ ion is stereochemically active and engenders a strong distortion of the local coordination environment, resulting in distinctive off-centering and reduction in local symmetry for the Tl ions, which are not observed for its s-block monovalent counterpart. HAXPES measurements corroborated by COHP calculations reveal that the local distortion is underpinned by hybridization of filled 6s^2^ and O 2p states, mediated by empty Tl 6p states, resulting in the formation of distinctive bonding states deep in the valence band and antibonding states at the valence band maximum. The conduction band has been probed by V L- and O K-edge NEXAFS measurements and in both compounds primarily comprises V 3d states that are split by crystal field splitting resulting from hybridization with the O 2p states. The second-order Jahn–Teller distortion engendered by Tl 6s^2^ lone pair states results in the elongation of a pair of Tl–O bonds and, conversely, strengthens Tl–O interactions on the opposite side of the interstitial site. The off-centering of Tl ions within their coordination environments weakens V–O interactions along this direction, which brings adjacent vanadium atoms into closer proximity. The stronger V–V coupling increases the ferromagnetic ordering temperature to ca. 140 K for TlV_3_O_8_ as compared to 125 K for RbV_3_O_8_ with oxide ions serving as superexchange mediators between the square pyramidal and octahedral chains. The lone pair distortion thus modulates the oxide-mediated superexchange. In conclusion, these results demonstrate that the local crystallographic environment and, ultimately, superexchange-driven magnetic properties are strongly influenced by 6s^2^ stereochemically active lone pairs in TlV_3_O_8_, which are stabilized through anion hybridization mediated by a reduction in local symmetry. The energy positioning of lone-pair states at the valence band edge suggests possible applications in photocatalysis. Future work will focus on modulation of the energetics of the lone-pair state through incorporation of site-selective modification.

## Supporting Information Available

Crystal data and structure refinement parameters; atomic coordinates, occupancies, and equivalent isotropic displacement parameters; anisotropic displacement parameters, bond lengths and angles; fitting of HAXPES data; crystal morphology; EDS; core level HAXPES characterization; density of states plots; additional magnetic susceptibility and magnetization data; inverse magnetic susceptibility data, Honda–Owen representation (PDF)
