# Supplementary material for: Effect of Stereochemically Active Electron Lone Pairs on Magnetic Ordering in Trivanadates
Source: Inorg Chem. 2023 Aug 2;62(32):12965–75. doi: 10.1021/acs.inorgchem.3c01760 (PMC10862544; doi:10.1021/acs.inorgchem.3c01760)
Supplement: Supplementary file 1 — ic3c01760_si_001.pdf [file ic3c01760_si_001.pdf]

Supporting Information

Effect of Stereochemically Active Electron Lone Pairs on Magnetic Ordering in Trivanadates

George Agbeworvi,[†,‡] Wasif Zaheer,[†,‡] John D. Ponis,[†] Joseph V. Handy,[†] Jaime R. Ayala,[†] Justin L. Andrews,[†] Parker Schofield,[†] Cherno Jaye,[⊥] Conan Weiland,[⊥] Daniel A. Fischer,[⊥] Sarbajit Banerjee[†]*

† Department of Chemistry and Department of Material Science and Engineering, Texas A&M University, College Station, TX, 77845-3012, USA;

⊥ Material Measurement Laboratory, National Institute of Standards and Technology, Gaithersburg, MD, 20899, USA

'‡' Equal contribution

'*' Corresponding author

Corresponding Author's Email Address: banerjee@chem.tamu.edu

Table S1. Crystal data and structure refinement for TlV_3O_8: TlVO1_6541_0m_a.

**Identification code**	6541tlvo1	
**Empirical formula**	O8 Tl V3	
**Formula weight**	485.19	
**Temperature**	110.00 K	
**Wavelength**	0.71073 Å	
**Crystal system**	Monoclinic	
**Space group**	P 1 21/m 1	
**Unit cell dimensions**	*a* = 4.96990(10) Å	α= 90°.
	b = 8.3814(2) Å	β= 96.3250(10)°.
	c = 7.7318(2) Å	γ = 90°.
**Volume**	320.105(13) Å^3	
**Z**	2	
**Density (calculated)**	5.034 mg/m^3	
**Absorption coefficient**	29.340 mm^{-1}	
**F(000)**	428	
**Crystal size**	0.13 × 0.065 × 0.005 mm^3	
**Theta range for data collection**	4.126 to 45.355°	
**Index ranges**	-9<=h<=9, -16<=k<=16, -15<=l<=15	
**Reflections collected**	27467	
**Independent reflections**	2803 [R(int) = 0.0606]	
**Completeness to theta = 25.242°**	99.50%	
**Absorption correction**	Semi-empirical from equivalents	
**Max. and min. transmission**	0.3555 and 0.1742	

**Refinement method**	Full-matrix least-squares on F^2
**Data / restraints / parameters**	2803 / 0 / 61
**Goodness-of-fit on F^2**	1.061
**Final R indices [I>2sigma(I)]**	R1 = 0.0232, wR2 = 0.0477
**R indices (all data)**	R1 = 0.0313, wR2 = 0.0494
**Largest diff. peak and hole**	0.3555 and 0.1742

Table S2. Atomic coordinates, occupancies, and equivalent isotropic displacement parameters (Å^2) for TlV_3O_8: TlVO1_6541_0m_a. U(eq) is defined as one third of the trace of the orthogonalized U^{ij} tensor.

Atom	*x*	*y*	*z*	Occupancy	Uiso
Tl(1)	0.9493(1)	0.7500	1.0569(1)	1	0.012(1)
V(2)	0.4376(1)	0.5540(1)	0.6882(1)	1	0.004(1)
V(1)	0.9179(1)	0.7500	0.5767(1)	1	0.004(1)
O(5)	0.3126(3)	0.4143(2)	0.4948(2)	1	0.006(1)
O(3)	0.5666(3)	0.4324(2)	0.8341(2)	1	0.008(1)
O(4)	0.1137(2)	0.5986(2)	0.7398(2)	1	0.006(1)
O(2)	0.5924(3)	0.7500	0.7603(2)	1	0.006(1)
O(1)	0.11277(4)	0.7500	0.4328(2)	1	0.008(1)

Table S3. Anisotropic displacement parameters (Å^{2}x 10^3) for TlV_3O_8: TlVO1_6541_0m_a. The anisotropic displacement factor exponent takes the form: $-2\pi^2[h^2 a^{*2}U^{11} + ... + 2 h k a^* b^* U^{12}]$

Atom	U11	U22	U33	U23	U13	U12
Tl(1)	10(1)	17(1)	8(1)	0	0(1)	0
V(2)	4(1)	4(1)	4(1)	0(1)	0(1)	0(1)
V(1)	3(1)	5(1)	5(1)	0	0(1)	0
O(5)	6(1)	6(1)	6(1)	-2(1)	1(1)	-1(1)
O(3)	9(1)	8(1)	7(1)	1(1)	0(1)	2(1)
O(4)	5(1)	6(1)	6(1)	0(1)	0(1)	0(1)
O(2)	6(1)	6(1)	6(1)	0	0(1)	0
O(1)	7(1)	8(1)	8(1)	0	3(1)	0

Table S4. Bond lengths [Å] and angles [°] for TlV_3O_8: TlVO1_6541_0m_a.

Atom pairs	Bond Length [Å]	Atom pairs	Bond Length [Å]
Tl(1)—O(2)	2.7401(18)	V(2)—O(2)	1.8729(9)
V(2)—V(1)	3.0976(4)	V(1)—O(5)#2	1.8370(13)
V(2)—V(1)#1	3.1020(4)	V(1)—O(5)#3	1.8370(13)
V(2)—O(5)#2	2.0004(13)	V(1)—O(4)#4	1.9690(13)
V(2)—O(5)	1.9464(13)	V(1)—O(4)#5	1.9690(13)
V(2)—O(3)	1.6007(14)	V(1)—O(2)	2.2668(18)
V(2)—O(4)	1.7413(12)	V(1)—O(1)	1.6068(19)

Atoms	Bond Angle [°]	Atoms	Bond Angle [°]
V(1)—V(2)—V(1)#1	106.577(10)	O(5)#2—V(1)—O(4)#4	157.46(6)
O(5)—V(2)—V(1)	107.20(4)	O(5)#2—V(1)—O(4)#5	87.55(6)
O(5)#2—V(2)—V(1)	34.43(4)	O(5)#2—V(1)—O(2)	73.98(5)
O(5)—V(2—V(1)#1	85.39(4)	O(5)#3—V(1)—O(2)	73.98(5)
O(5)#2—V(2)—V(1)#1	107.20(4)	O(4)#5—V(1)—V(2)#5	31.03(4)
O(5)—V(2)—O(5)#2	72.96(6)	O(4)#4—V(1)—V(2)	120.34(4)
O(3) —V(2)—V(1)	105.81(5)	O(4)#4—V(1)—V(2)#4	31.03(4)
O(3)—V(2)—V(1)#1	142.16(5)	O(4)#5—V(1)—V(2)#6	120.34(4)
O(3)—V(2)—O(5)	103.16(7)	O(4)#4—V(1)—V(2)#5	79.98(4)
O(3)—V(2)—O(5)#2	110.56(6)	O(4)#5—V(1)—V(2)#4	79.98(4)
O(3)—V(2)—O(4)	106.51(7)	O(4)#4—V(1)—V(2)#6	79.71(4)
O(3—V(2—O(2)	103.25(7)	O(4)#5—V(1)—V(2)	79.71(4)
O(4)—V(2)—V(1)	135.25(5)	O(4)#4—V(1)—O(4)#5	80.26(8)
O(4—V(2)—V(1)#1	35.66(4)	O(4)#4—V(1)—O(2)	86.29(5)
O(4)—V(2)—O(5)#2	142.66(6)	O(4)#5—V(1)—O(2)	86.29(5)
O(4)—V(2)—O(5)	94.61(6)	O(2)—V(1)—V(2)#4	117.12(4)
O(4)—V(2)—O(2)	95.93(7)	O(2)—V(1)—V(2)#5	117.12(4)
O(2)—V(2)—V(1)	46.65(5)	O(2)—V(1)—V(2)	36.93(2)
O(2)—V(2—V(1)#1	85.42(5)	O(2)—V(1)—V(2)#6	36.93(2)
O(2)—V(2)—O(5)	147.37(7)	O(1)—V(1)—V(2)	140.49(4)
O(2)—V(2) —O(5)#2	80.05(7)	O(1)—V(1)—V(2)#4	66.96(6)
V(2)—V(1)—V(2)#5	106.577(10)	O(1)—V(1)—V(2)#6	140.49(4)
V(2)—V(1)—V(2)#4	147.909(17)	O(1)—V(1)—V(2)#5	66.96(6)
V(2)—V(1)—V(2)#6	64.069(13)	O(1)—V(1)—O(5)#3	102.84(6)
V(2)#6—V(1)—V(2)#5	147.909(17)	O(1)—V(1)—O(5)#2	102.84(6)
V(2)#6—V(1)—V(2)#4	106.577(10)	O(1)—V(1)—O(4)#5	97.54(6)
V(2)#4—V(1)—V(2)#5	63.967(13)	O(1)—V(1)—O(4)#4	97.54(7)
O(5)#2—V(1)—V(2)#4	162.40(4)	O(1)—V(1)—O(2)	174.97(8)
O(5)#2—V(1)—V(2)#6	90.41(4)	V(2)—O(5)—V(2)#2	107.04(6)
O(5)#2—V(1)—V(2)#5	99.15(4)	V(1)#2—O(5)—V(2)#2	107.57(6)
O(5)#3—V(1)—V(2)#4	99.15(4)	V(1)#2—O(5)—V(2)	144.86(8)
O(5)#3—V(1)—V(2)#6	38.00(4)	V(2)—O(4)—V(1)#1	113.31(7)
O(5)#3—V(1)—V(2)	90.41(4)	V(2)—O(2)—Tl(1)	117.12(5)
O(5)#2—V(1)—V(2)	38.00(4)	V(2)#6—O(2)—Tl(1)	117.12(5)
O(5)#3—V(1) —V(2)#5	162.40(4)	V(2)—O(2)—V(2)#6	122.63(10)

O(5)#2—V(1)—O(5)#3	97.11(8)	V(2)—O(2)—V(1)	96.42(6)
O(5)#3—V(1)—O(4)#5	157.46(6)	V(2)#6—O(2)—V(1)	96.42(6)
O(5)#3—V(1)—O(4)#4	87.55(6)	V(1)—O(2)—Tl(1)	94.77(6)

Symmetry transformations used to generate equivalent atoms:

#1 x-1,y,z #2 -x+1,-y+1,-z+1 #3 -x+1,y+1/2,-z+1

#4 x+1,-y+3/2,z #5 x+1,y,z #6 x,-y+3/2,z

Table S5. Crystal data and structure refinement for RbV_3O_8: Rb3v3o8_ 31o1_1_x1_110k_0m_5.

**Identification code**	Rb3v3o8_ 31o1_1_x1_110k_0m_5	
**Empirical formula**	Rb O8 V3	
**Formula weight**	366.29	
**Temperature**	110(2) K	
**Wavelength**	0.71084 Å	
**Crystal system**	Monoclinic	
**Space group**	P 21/m	
**Unit cell dimensions**	a = 4.9729(4) Å	α= 90°.
	b = 8.4106(7) Å	β= 95.783(3)°.
	c = 7.8171(7) Å	γ = 90°.
**Volume**	325.29(5) $Å^3$	
**Z**	2	
**Density (calculated)**	3.739 mg/m^3	
**Absorption coefficient**	11.657 mm^{-1}	
**F(000)**	340	
**Crystal size**	0.154 × 0.143 × 0.010 mm^3	
**Theta range for data collection**	2.619 to 40.092°.	
**Index ranges**	-8<=h<=8, -14<=k<=14, -13<=l<=13	
**Reflections collected**	9589	
**Independent reflections**	1947 [R(int) = 0.0802]	
**Completeness to theta = 25.242°**	100.00%	
**Absorption correction**	multi-scan	
**Max. and min. transmission**	0.7479 and 0.4119	
**Refinement method**	Full-matrix least-squares on F^2	
**Data / restraints / parameters**	1947 / 0 / 61	
**Goodness-of-fit on F^2**	1.048	
**Final R indices [I>2sigma(I)]**	R1 = 0.0532, wR2 = 0.1344	
**R indices (all data)**	R1 = 0.0684, wR2 = 0.1471	
**Largest diff. peak and hole**	1.479 and -2.368 $e.Å^{-3}$	

Table S6. Atomic coordinates, occupancies, and equivalent isotropic displacement parameters ($Å^2$) for RbV_3O_8: Rb3v3o8_31o1_1_x1_110k_0m_5. U(eq) is defined as one third of the trace of the orthogonalized U^{ij} tensor.

Atom	x	y	z	Occupancy	Uiso
Rb(1)	0.9452(10)	0.7500	1.0643(7)	1	0.013(13)
V(1)	0.848(16)	0.2500	0.4239(12)	1	0.008(15)
V(2)	0.4345(11)	0.5547(7)	0.5547(7)	1	0.008(13)
O(1)	-0.1250(8)	0.2500	0.5680(6)	1	0.011(6)
O(2)	0.3135(5)	0.4136(3)	0.4951(4)	1	0.010(4)
O(3)	0.5584(6)	0.4323(4)	0.8311(4)	1	0.012(5)
O(4)	0.5884(7)	0.7500	0.7561(5)	1	0.009(6)
O(5)	0.1110(5)	0.5984(3)	0.7366(4)	1	0.010(4)

Table S7. Anisotropic displacement parameters ($Å^2$x 10^3) for RbV_3O_8: Rb3v3o8_ 31o1_1_x1_110k_0m_5. The anisotropic displacement factor exponent takes the form: $-2\pi^2[h^2 a^{*2}U^{11} + ... + 2 h k a^* b^* U^{12}]$

Atom	U11	U22	U33	U23	U13	U12
Rb(1)	9(2)	16(2)	14(2)	0	1(15)	0
V(1)	5(3)	7(3)	12(3)	0	2(2)	0
V(2)	6(2)	7(2)	12(2)	1(18)	2(17)	0(15)
O(1)	8(13)	10(15)	16(17)	0	3(12)	0
O(2)	7(9)	8(10)	16(11)	-2(9)	1(8)	0(7)
O(3)	10(10)	12(11)	14(11)	3(9)	1(8)	1(8)
O(4)	6(12)	6(13)	15(16)	0	0(11)	0
O(5)	7(9)	15(11)	15(11)	-1(8)	2(8)	0(7)

Table S8. Bond lengths [Å] and angles [°] for RbV_3O_8: RbV_3O_8: Rb3v3o8_ 31o1_1_x1_110k_0m_5.

Atoms	Bond Length [Å]	Atoms	Bond Length [Å]
V(1)—O(4)#4	2.254(4)	V(2)—O(4)	1.8710(19)
V(1)—O(5)#2	1.975(3)	V(2)—O(5)	1.734(3)
V(1)—O(5)#3	1.975(3)	V(2)—O(2)#4	2.001(3)
V(1)—O(2)#5	1.836(3)	V(2)—O(2)	1.953(3)
V(1)—O(2)	1.836(3)	V(2)—O(3)	1.609(3)
V(1)—O(1)	1.610(4)	V(2)—V(1)	3.1080(9)

Atoms	Bond Angle [°]	Atoms	Bond Angle [°]
V(2)#3—V(1)—V(2)#2	63.83(3)	O(2)#5—V(1)—O(5)#3	87.66(12)
V(2)#1—V(1)—V(2)#3	106.63(2)	O(2)#5—V(1)—O(5)#2	157.99(13)
V(2)#4—V(1)—V(2)#3	147.97(4)	O(2)—V(1)—O(2)#5	97.14(18)
V(2)#4—V(1)—V(2)#1	64.17(3)	O(2)—V(1)—O(2)#1	140.14(9)
V(2)#1—V(1)—V(2)#2	147.97(4)	V(1)#4—V(2)—V(1)#2	106.63(2)
V(2)#4—V(1)—V(2)#2	106.63(2)	O(4)—V(2)—V(1)#2	85.45(10)
O(4)#4—V(1)—V(2)#2	117.29(8)	O(4)—V(2)—V(1)#4	46.37(12)
O(4)#4—V(1)—V(2)#1	36.92(5)	O(4)—V(2)—V(2)	147.37(16)
O(4)#4—V(1)—V(2)#4	36.92(5)	O(4)—V(2)—O(2)#4	79.81(15)
O(4)#4—V(1)—V(2)#3	117.29(8)	O(5)—V(2)—V(1)#2	35.6(1)
O(5)#2—V(1)—V(2)#4	79.98(8)	O(5)—V(2)—V(1)#4	135.34(10)
O(5)#3—V(1)—V(2)#2	79.80(8)	O(5)—V(2)—O(4)	96.23(14)
O(5)#2—V(1)—V(2)#2	30.74(8)	O(5)—V(2)—O(2)#4	142.59(13)
O(5)#2—V(1)—V(2)#1	120.76(9)	O(5)—V(2)—O(2)	94.67(12)
O(5)#3—V(1)—V(2)#4	120.76(9)	O(2)—V(2)—V(1)#4	107.29(9)
O(5)#3—V(1)—V(2)#3	30.74(8)	O(2)#4—V(2)—V(1)#2	107.21(8)
O(5)#2—V(1)—V(2)#3	79.80(8)	O(2)#4—V(2)—V(1)#4	34.50(8)
O(5)#3—V(1)—V(2)#1	79.86(8)	O(2)—V(2)—V(1)#2	85.77(8)
O(5)#3—V(1)—O(4)#4	86.77(11)	O(2)—V(2)—O(2)#4	72.95(13)
O(5)#2—V(1)—O(4)#4	86.77(11)	O(3)—V(2)—V(1)#2	141.64(11)
O(5)#3—V(1)—O(5)#2	80.41(16)	O(3)—V(2)—V(1)#4	106.34(11)
O(2)—V(1)—V(2)#4	38.13(9)	O(3)—V(2)—O(4)	103.78(16)
O(2)#5—V(1)—V(2)#3	162.24(9)	O(3)—V(2)—O(5)	102.55(14)
O(2)—V(1)—V(2)#3	162.24(9)	O(3)—V(2)—O(2)#4	111.05(14)
O(2)—V(1)—V(2)#2	99.19(9)	V(2)—O(4)—V(1)#4	96.71(14)
O(2)#5—V(1)—V(2)#3	99.19(9)	V(2)#6—O(4)—V(1)#4	96.70(14)
O(2)#5—V(1)—V(2)#4	90.57(9)	V(2)—O(4)—V(2)#6	122.8(2)
O(2)#5—V(1)—V(2)#1	38.13(9)	V(2)—O(5)—V(1)#2	113.66(15)
O(2)—V(1)—V(2)#1	90.57(9)	V(1)—O(2)—V(2)	145.14(17)
O(2)—V(1)—O(4)#4	74.07(11)	V(1)—O(2)—V(2)#4	107.37(14)
O(2)#5—V(1)—O(4)#4	74.07(11)	V(2)—O(2)—V(2)#4	107.05(12)
O(2)—V(1)—O(5)#2	87.66(12)		
O(2)—V(1)—O(5)#3	157.99(13)		

Symmetry transformations used to generate equivalent atoms:

#1 1-x, -0.5+y, 1-z; #2 -x, 1-y, 1-z; #3 -x, -0.5+y, 1-z; #4 1-x, 1-y, 1-z;

#5 x, 0.5-y, z; #6 x, 1.5-y, z.

Table S9: Fitting of HAXPES data for V 2p and O 1s as obtained for RbV_3O_8 and plotted in Figure S4.

**V2*p*_O1*s* RbV_3O_8**			
Core Level (Oxidation State)	Position (eV)	FWHM (eV)	% Area
V $2p_{3/2}$ (IV)	515.70	1.593	3.74
V $2p_{3/2}$ (V)	517.14	1.400	27.29
V $2p_{1/2}$ (IV)	522.94	1.535	1.32
V $2p_{1/2}$ (V)	524.61	2.057	9.47
O 1s	530.00	1.500	47.73
O 1s	531.53	1.500	7.16
O 1s water/organic	533.13	1.500	3.30

% of V^{4+} in RbV_3O_8 = Percentage Area (V^{4+}) / [Percentage Area (V^{4+}) + Percentage Area (V^{5+})]

= 3.74 + 1.32 /(3.74 +1.32 + 27.29 + 9.47) = 12.1%

% of V^{5+} in RbV_3O_8 = 100 – % of V^{4+} in RbV_3O_8 = 100 – 12.1 % = 87.9%

Table S10: Fitting of HAXPES data for V 2p and O 1s as obtained for TlV_3O_8 and plotted in Figure S4.

**V2*p*_O1*s* TlV_3O_8**			
Core Level (Oxidation State)	Position (eV)	FWHM (eV)	% Area
V $2p_{3/2}$ (IV)	516.00	1.470	3.46
V $2p_{3/2}$ (V)	517.03	1.205	27.16
V $2p_{1/2}$ (IV)	522.79	1.797	1.38
V $2p_{1/2}$ (V)	524.48	2.000	10.06
O 1s	530.00	1.221	49.47
O 1s	531.03	1.202	6.13
O 1s water/organic	532.49	1.500	2.34

% of V^{4+} in TlV_3O_8 = Percentage Area (V^{4+}) / [Percentage Area (V^{4+}) + Percentage Area (V^{5+})]

= 3.46 + 1.38 / (3.46 + 1.38 + 27.16 + 10.06) = 11.5%

% of V^{5+} in TlV_3O_8 = 100 – % of V^{4+} in TlV_3O_8 = 100 –11.5 = 88.5%

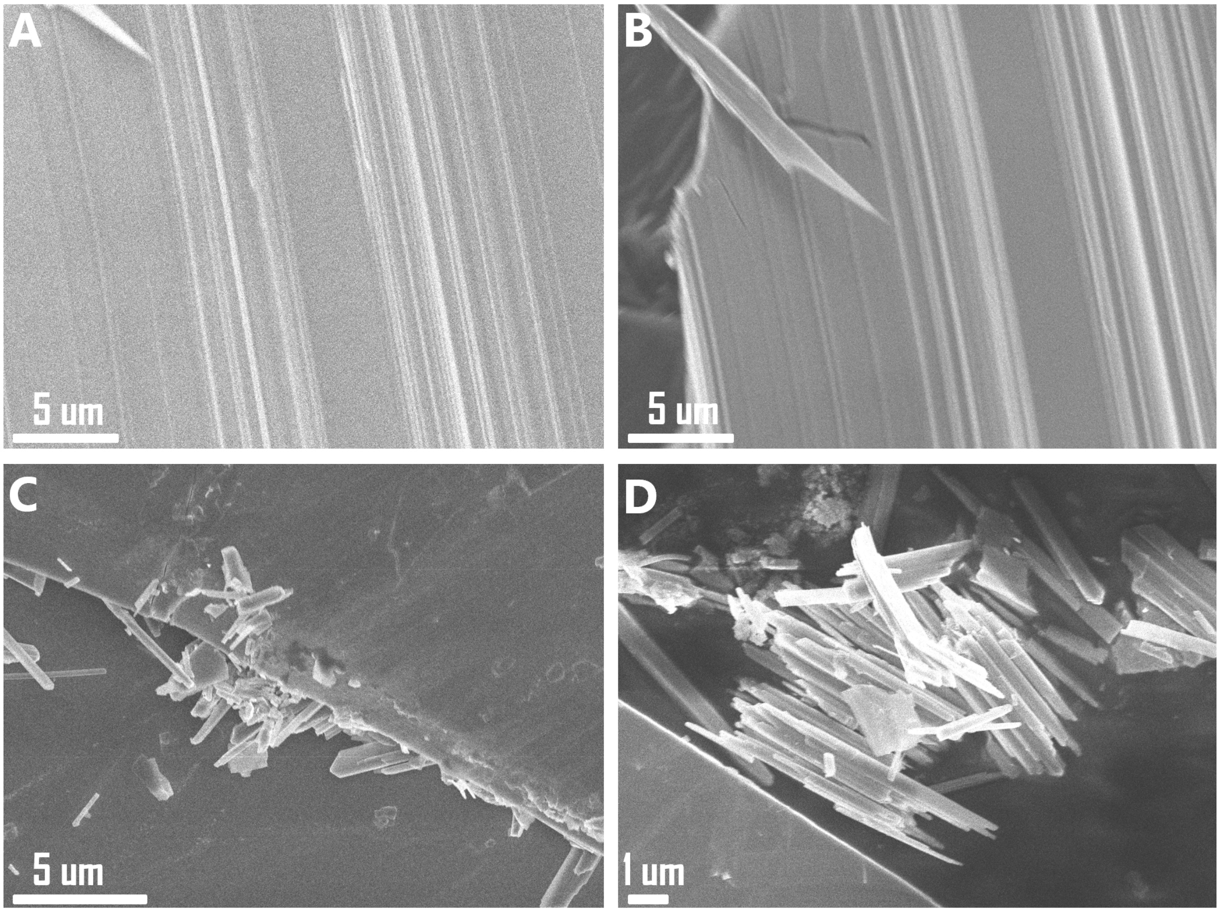

***Figure S1. Crystal morphology**. SEM images acquired for RbV_3O_8 **(A, B)** and TlV_3O_8 **(C, D)**.*

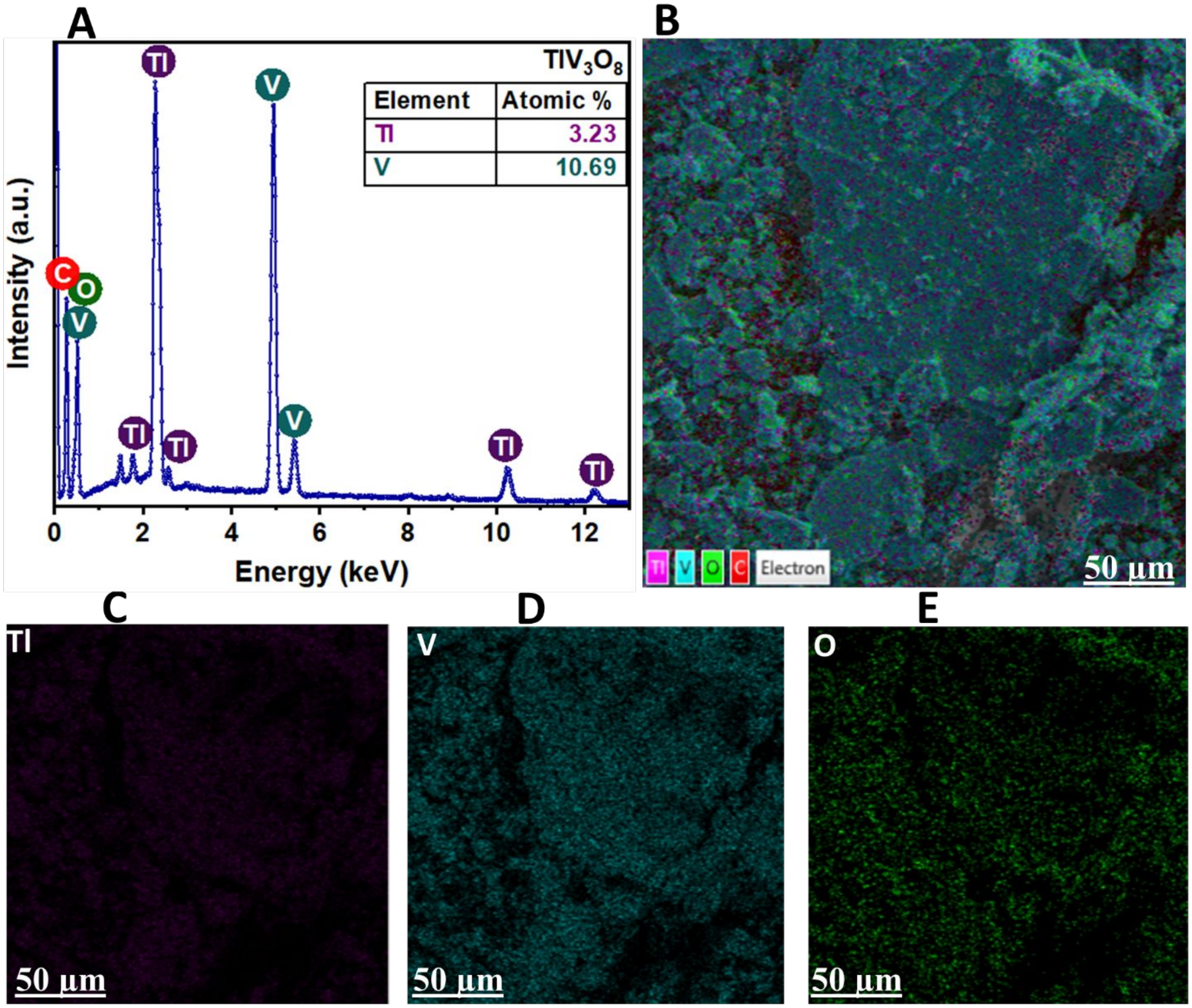

Figure S2. Energy dispersive X-ray spectroscopy mapping analysis of TlV_3O_8. *A) Energy dispersive X-ray spectrum acquired for TlV_3O_8. The inset table (top right) shows the elemental composition of TlV_3O_8 as measured by EDX. Elemental mapping showing B) superimposed maps and spatial distribution of C) Tl, D) V, and E) O.*

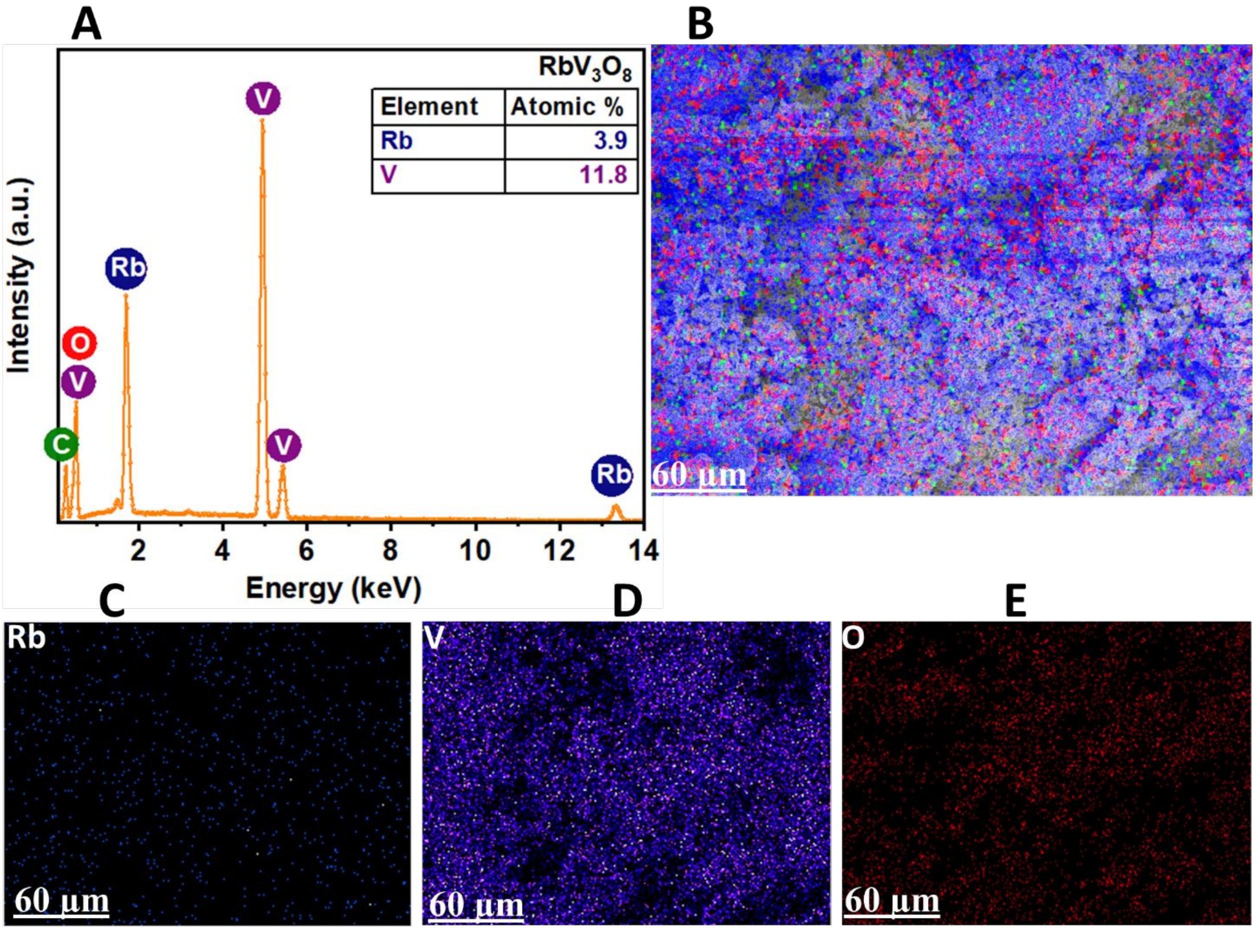

Figure S3. Energy dispersive X-ray spectroscopy mapping analysis of RbV_3O_8. *A) Energy dispersive X-ray spectrum acquired for RbV_3O_8. The inset table (top right) shows the elemental composition of RbV_3O_8 as measured by EDX. Elemental mapping showing B) superimposed maps and spatial distribution of C) Tl, D) V, and E) O.*

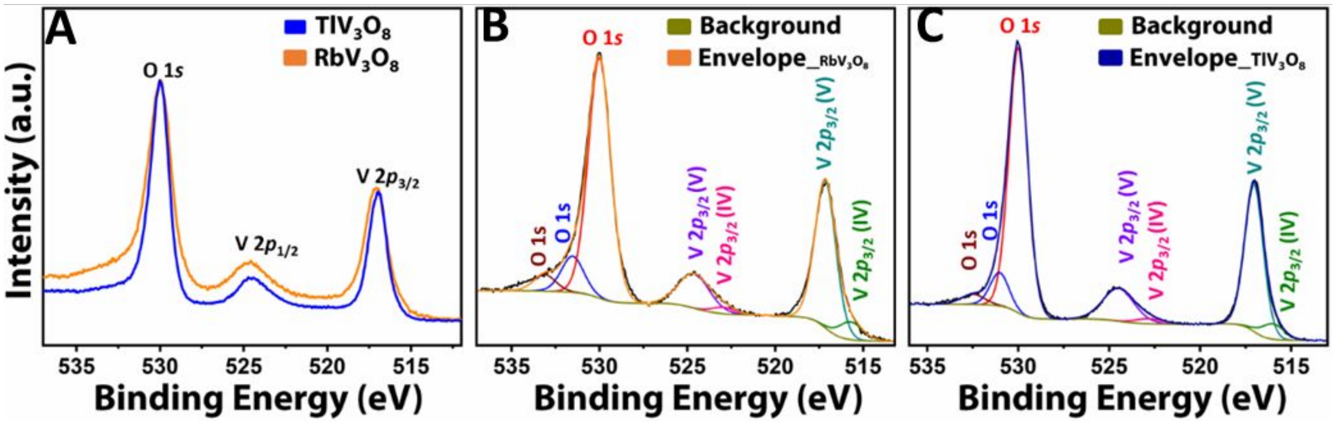

Figure S4. Core level HAXPES characterization. (A) *O 1s and V 2p high-resolution HAXPES plots acquired at an incident energy of 2 keV for RbV_3O_8 and TlV_3O_8. Fitting of HAXPES data for* ***(B)*** *RbV_3O_8 and* ***(C)*** *TlV_3O_8.*

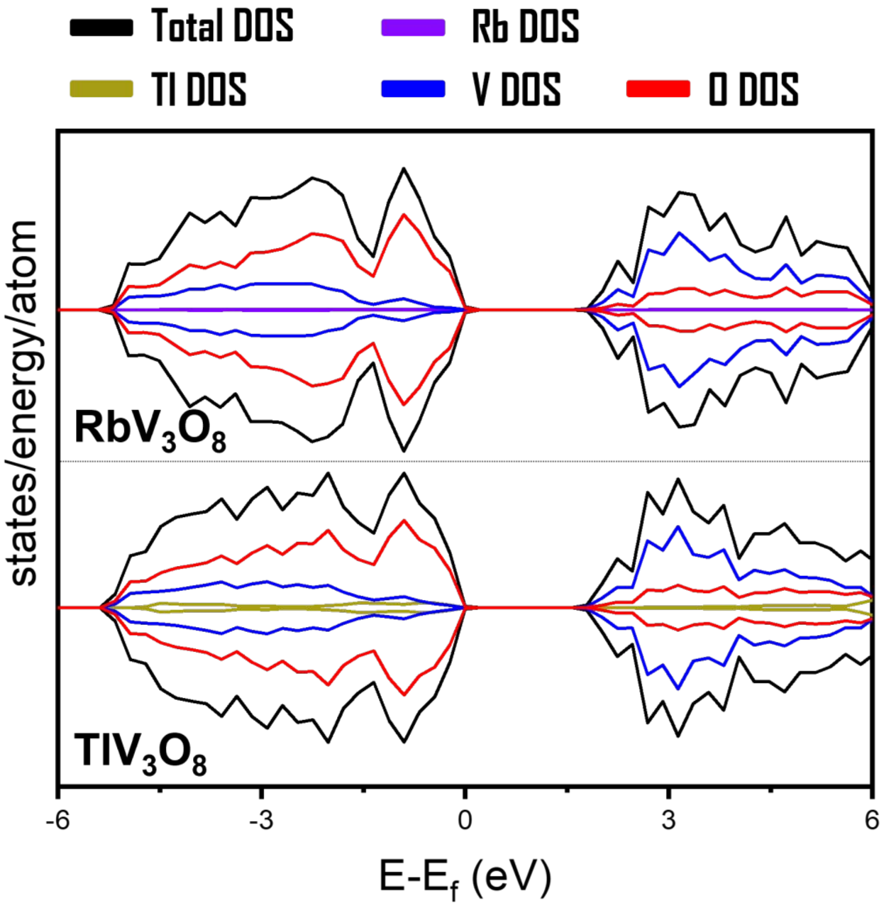

Figure S5. Calculated density of states comparison. *Comparison plot of ground-state density of states (DOS) for* RbV_3O_8 *and* TlV_3O_8 *as calculated by the GGA+U method. The valence band maximum is aligned to the Fermi level.*

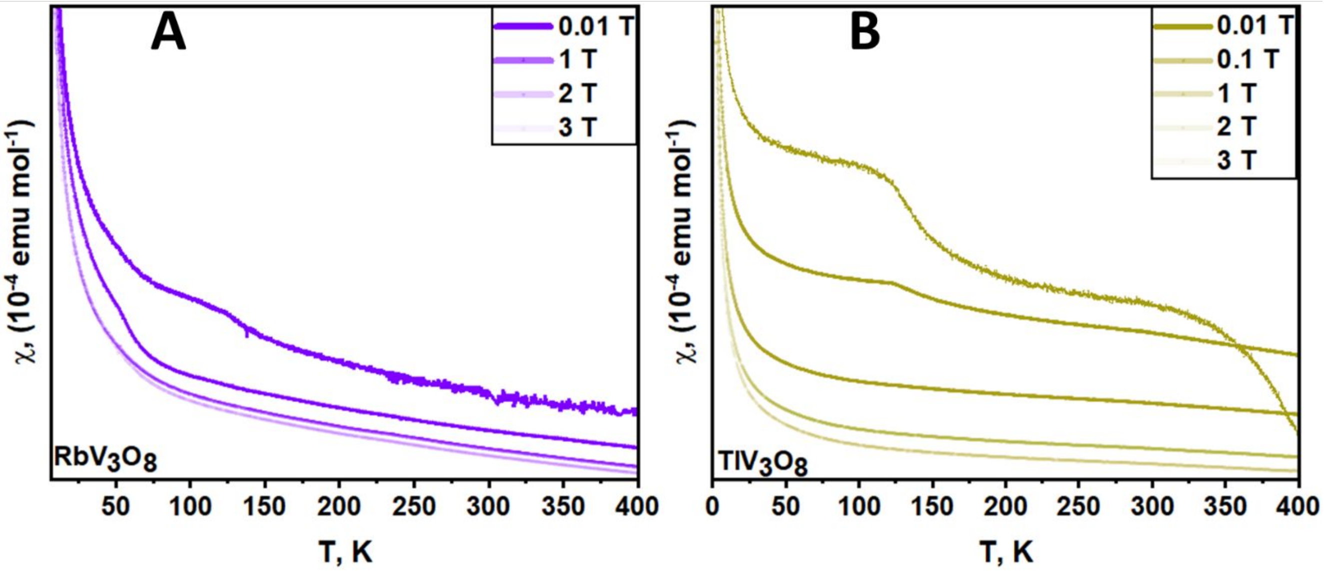

Figure S6. Magnetic susceptibility measurements at varying field strengths. *FC magnetic susceptibility of (A)* RbV_3O_8 *and (B)* TlV_3O_8 *at* $\mu_0(H)$ *= 0.01, 0.1, 1, 2, and 3T.*

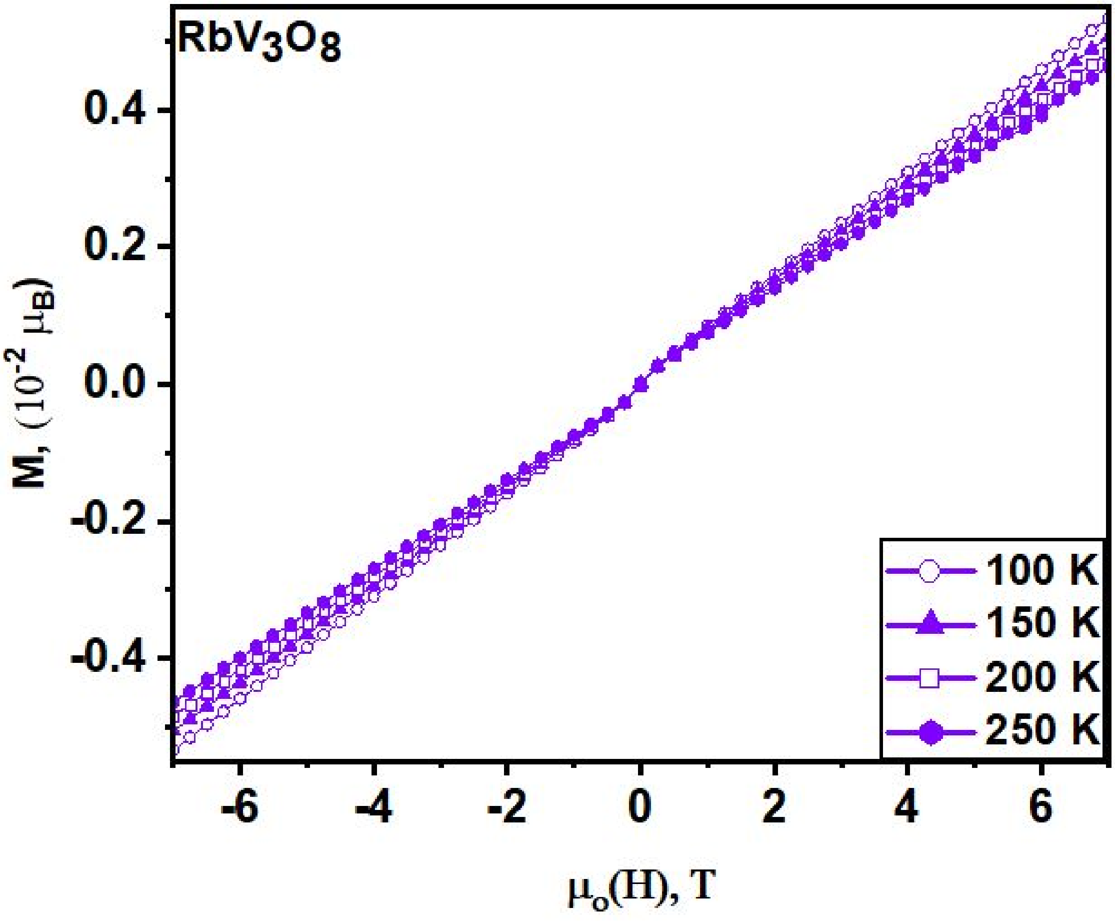

Figure S7*. *Magnetization of RbV_3O_8 ***at varying temperatures***. *Magnetization versus magnetic field curve plotted at 100, 150, 200, and 250 K for* RbV_3O_8.

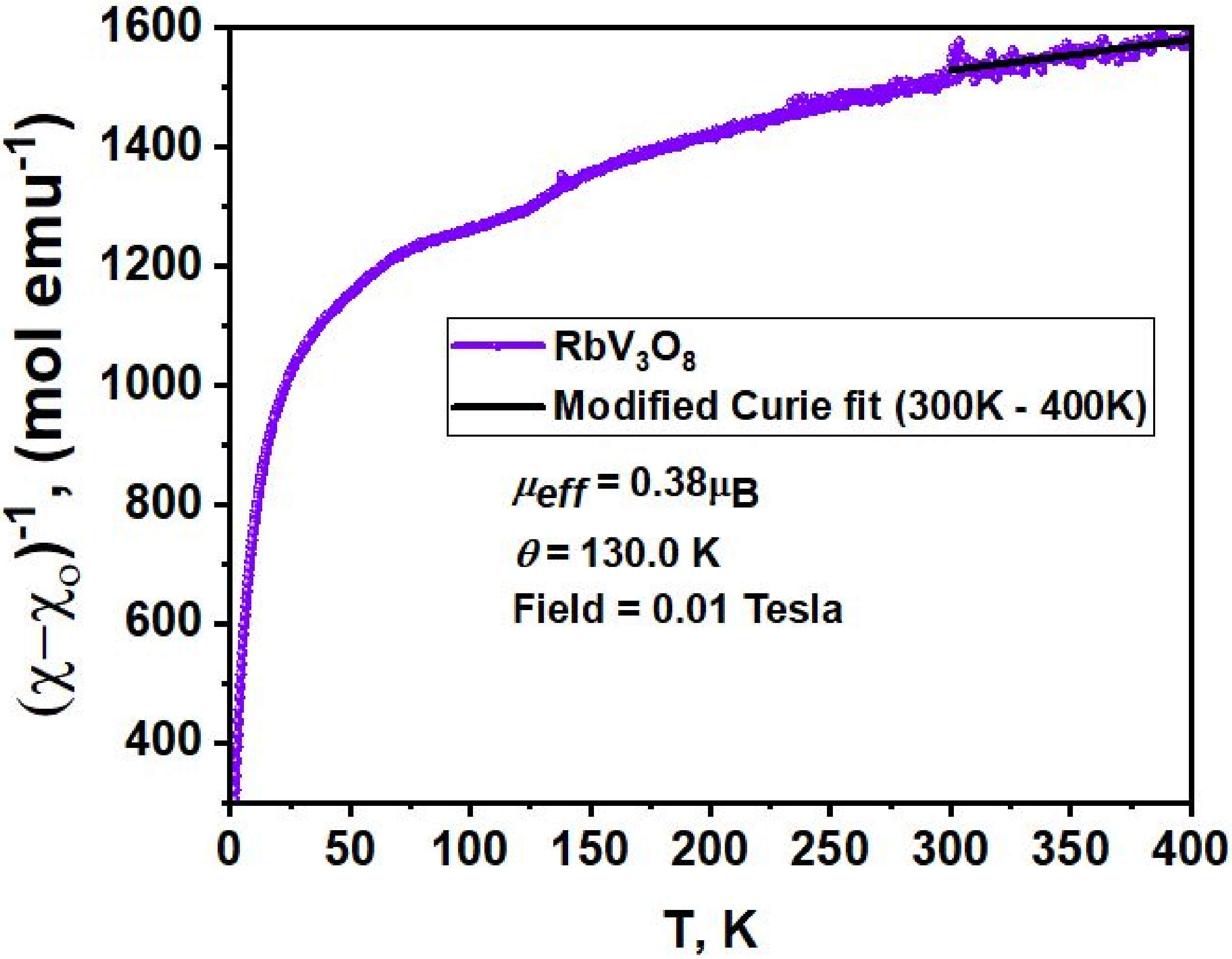

***Figure S8**. **Inverse magnetic susceptibility of RbV_3O_8.** Reciprocal plot of the magnetic susceptibility data at 2−400 K and results of the Modified Curie−Weiss fitting for RbV_3O_8.*

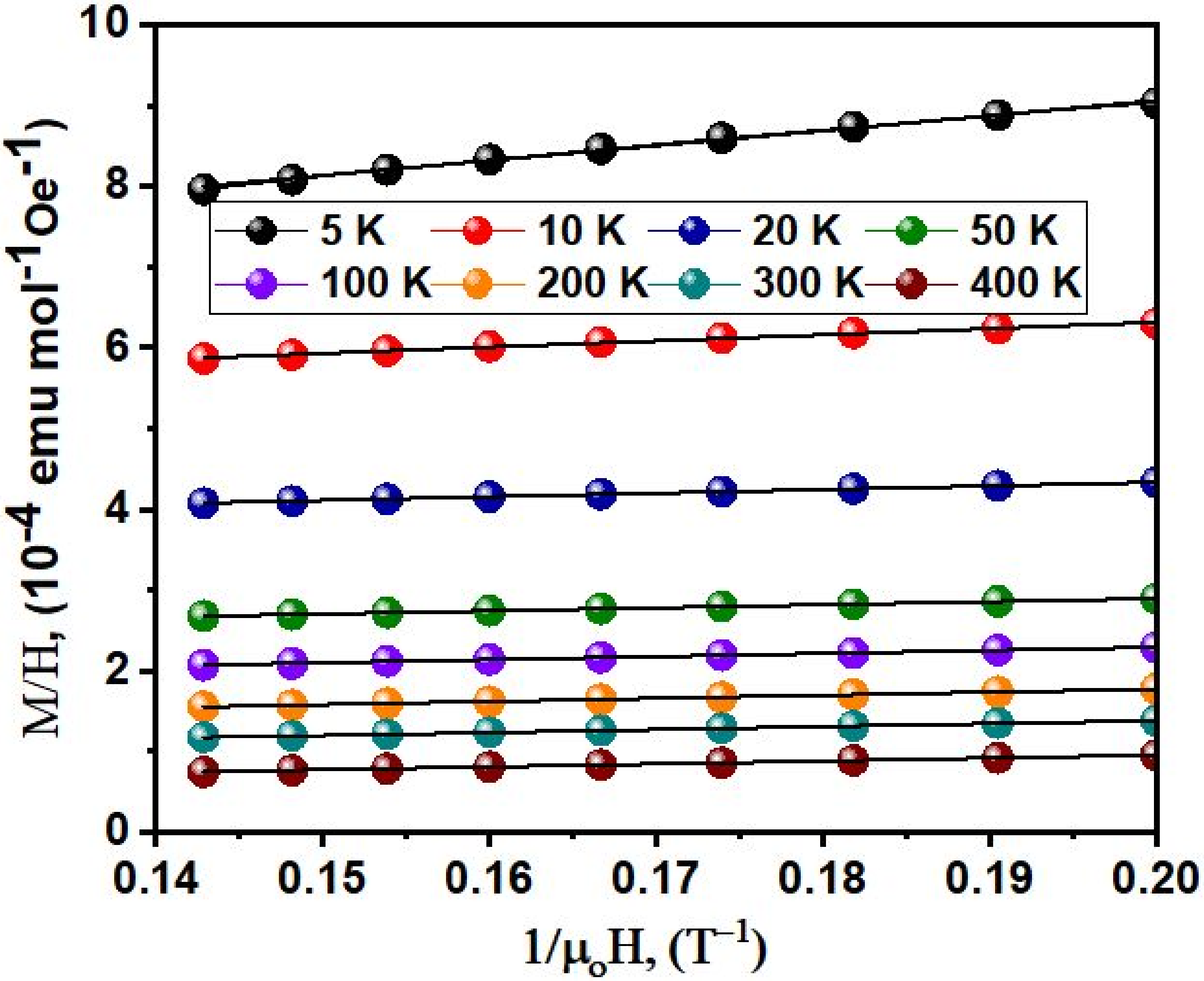

***Figure S9**. Field dependence of magnetization of TlV_3O_8 shown in a Honda-Owen representation*. *Field-dependent magnetization of TlV_3O_8 between 5 and 7 T at 5, 10, 20, 50, 100, 200, 300 and 400 K. The data (M/H) are plotted as a function of 1/H at higher applied fields according to the Honda-Owen representation.*
